# Proton gradients from light-harvesting *E. coli* control DNA assemblies for synthetic cells

**DOI:** 10.1038/s41467-021-24103-x

**Published:** 2021-06-25

**Authors:** Kevin Jahnke, Noah Ritzmann, Julius Fichtler, Anna Nitschke, Yannik Dreher, Tobias Abele, Götz Hofhaus, Ilia Platzman, Rasmus R. Schröder, Daniel J. Müller, Joachim P. Spatz, Kerstin Göpfrich

**Affiliations:** 1 Biophysical Engineering Group, Max Planck Institute for Medical Research, Heidelberg, Germany; 2 Department of Physics and Astronomy, Heidelberg University, Heidelberg, Germany; 3 Department of Biosystems Science and Engineering, Eidgenössische Technische Hochschule (ETH) Zurich, Basel, Switzerland; 4 Centre for Advanced Materials, Heidelberg, Germany; 5 Max Planck Institute for Medical Research, Department of Cellular Biophysics, Heidelberg, Germany; 6 Institute for Molecular Systems Engineering (IMSE), Heidelberg University, Heidelberg, Germany; 7 Max Planck School Matter to Life, Heidelberg, Germany

**Keywords:** Synthetic biology, Light harvesting, DNA nanostructures, Biological physics

## Abstract

Bottom-up and top-down approaches to synthetic biology each employ distinct methodologies with the common aim to harness living systems. Here, we realize a strategic merger of both approaches to convert light into proton gradients for the actuation of synthetic cellular systems. We genetically engineer *E. coli* to overexpress the light-driven inward-directed proton pump xenorhodopsin and encapsulate them in artificial cell-sized compartments. Exposing the compartments to light-dark cycles, we reversibly switch the pH by almost one pH unit and employ these pH gradients to trigger the attachment of DNA structures to the compartment periphery. For this purpose, a DNA triplex motif serves as a nanomechanical switch responding to the pH-trigger of the *E. coli*. When DNA origami plates are modified with the pH-sensitive triplex motif, the proton-pumping *E. coli* can trigger their attachment to giant unilamellar lipid vesicles (GUVs) upon illumination. A DNA cortex is formed upon DNA origami polymerization, which sculpts and deforms the GUVs. We foresee that the combination of bottom-up and top down approaches is an efficient way to engineer synthetic cells.

## Abstract (web summary)

Controlled actuation is an important aspect of synthetic cellular systems. Here, the authors combine pH responsive DNA origami structures with light triggered proton pump engineered *E. coli* to trigger a change in pH and control the deformation of giant unilamellar vesicles by simple illumination.

## Introduction

Synthetic biology cultivates an engineering approach to biology with the aim to create or to re-purpose biological parts for specific tasks. The field is commonly divided into two branches with distinct tools and methodologies, but also distinct challenges—top-down and bottom-up synthetic biology^1,2^. The top-down approach uses genetic engineering techniques to manipulate natural cells, reprogramming their behavior and equipping them with unique and exciting functions^3^. *Escherichia coli* (*E. coli*) bacteria, for instance, have been engineered for a variety of tasks, including biofuel production^4^, cancer cell targeting^5^ or light harvesting^6,7^. Yet living cells remain too complex to achieve full control and not all added functions are compatible with the host^8^.

The bottom-up approach, on the other hand, has been successful at reconstituting natural biomolecules, or artificial components in cell-sized confinement like microfluidic droplets or lipid vesicles^9–11^. Noteworthy modules have been implemented so far, each mimicking a specific function of a living cell, including energy generation^12,13^, metabolism^14^, motility^15,16^, cytoskeletal contraction^17^ or division^18^. Yet the combination of these modules towards complex signaling pathways for dynamic systems remains challenging. Merging the capacities of top-down and bottom-up approaches to synthetic biology can be a leap forward towards complex bottom-up assemblies but also more versatile and well-defined top-down systems. Leading to this direction, communication between natural and synthetic cells has been implemented^19–21^ and bottom-up assembled vesicles were used as organelle mimics in living cells^22^. Furthermore, engineered prokaryotes have recently been used as artificial organelles in living cells^23,24^, yet this has never been translated into synthetic cells.

Here, we use top-down genetic engineering to equip *E. coli* with light-harvesting capabilities. We employ them as synthetic organelle mimics inside bottom-up assembled synthetic cellular compartments. Thereby, we can reversibly switch the pH upon illumination to trigger an optical or a mechanical response. The latter is based on the pH-sensitive membrane attachment of a triplex-forming DNA motif triggered by proton gradients from light-harvesting *E. coli*. Furthermore, we employ the pH-gradients to sculpt synthetic cellular compartments by attaching a DNA origami plate to the pH-sensitive DNA strand.

## Results

### Top-down engineering of *E. coli* for light-harvesting

**Fig. 1:**
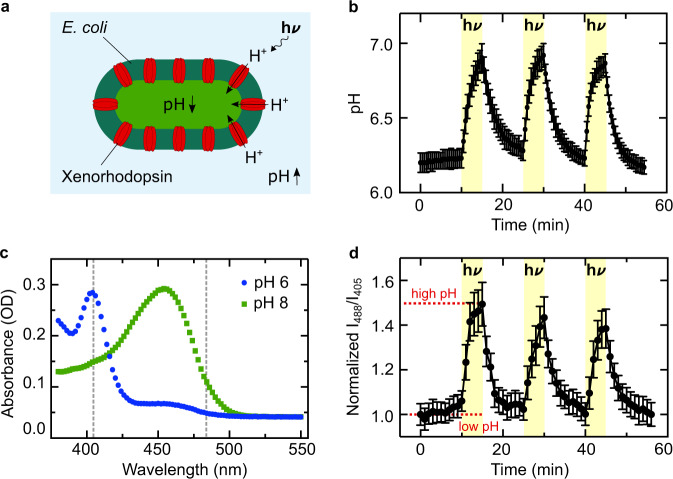
Genetically engineered xenorhodopsin-expressing *E. coli* generate a pH gradient upon illumination with white light. **a** Schematic illustration of an *E. coli* expressing xenorhodopsin, a light-driven proton pump (red), allowing for the reversible generation of a directional pH gradient during illumination with white light. The inward pump increases the pH of the external solution. **b** Photoactivity generated by the *E. coli* (OD_600_ =20, in 150 mM NaCl) measured with an external pH electrode. The pH is plotted over time during three light-dark cycles (periods of illumination are indicated in yellow). The pH increases by almost one pH unit within 5 min of illumination and nearly returns to its original value after 10 min in the dark (mean ± s.d., *n* = 3). **c** Absorbance measurements of the pH-sensitive ratiometric fluorophore pyranine at pH 6 (blue) and pH 8 (green). The pH can be quantified as the fluorescence intensity ratio at the excitation wavelengths 488 nm and 405 nm (gray dashed lines). **d** Normalized fluorescence intensity ratio I_488_/I_405_ of pyranine (50 μM) over time in a solution containing *E. coli* and lipid vesicles as determined with confocal fluorescence microscopy (mean ± s.d., *n* = 4). Periods of illumination are indicated in yellow. Source data is available for Fig. 1b–d.

To equip synthetic cells with the capability to generate proton gradients, we set out to assemble an energy module. We genetically engineered *E. coli* to overexpress the light-driven proton pump xenorhodopsin, a transmembrane protein from nanohalosarchaeon *Nanosalina*^25^. It contains a retinal which, upon illumination, undergoes a trans-cis conformational change and shuttles a proton across the lipid membrane. We chose xenorhodopsin because it shows unique features compared to other proton pumps, such as bacteriorhodopsin or proteorhodopsin: First of all, xenorhodopsin exhibits a substantially faster photocycle, which can result in larger proton gradients^25^. Second, as an inward-directed pump^26^, xenorhodopsin increases the pH (instead of decreasing it) in the extracellular space upon illumination (Fig. 1a). As an additional feature, we introduced a C-terminal fluorescent GFP or mCherry tag to xenorhodopsin for vizualization of the *E. coli*. The choice of two dyes allows us to work with different combinations of fluorophores as required.

To assess and quantify the proton pumping capabilities of the genetically engineered *E. coli*, we performed a photoactivity assay, where we inserted a micro pH electrode into the *E. coli* suspension and exposed it to multiple light-dark cycles. Since the absorption spectrum of xenorhodopsin covers a broad range, the use of a white light lamp is more effective than excitation with a specific wavelength (Supplementary Fig. 1). Illumination increased the pH in the extracellular space by almost one pH unit within five minutes (Fig. 1b), because protons are translocated from the extracellular solution to the cytosol. Longer illumination times resulted in saturation of the pH change (Supplementary Fig. 2). In the range from OD_600_ = 8 to OD_600_ = 40, the *E. coli* concentration did not significantly change the obtained pH gradients and we observed only a very minor increase in the kinetics at higher concentrations (Supplementary Fig. 3). The pH quickly returned to its initial value after the light was turned off due to the dissipation of protons. Even after three complete light-dark cycles, we observed only little decrease in the pH gradient. Compared to previous reports where proton pumps were reconstituted in lipid vesicles^7,27^, we could achieve faster and higher pH gradients using genetically engineered *E. coli*. Moreover, the use of *E. coli* circumvented the need for cumbersome protein purification and reconstitution to prepare proteoliposomes^28^, which highlights a key advantage of merging top-down and bottom-up synthetic biology.

As a next step, we aimed to encapsulate the *E. coli* as a pH switch in synthetic cells, which makes pH monitoring with an electrode impractical. We thus supplement the *E. coli* suspension with the ratiometric pH-sensitive fluorescent dye pyranine. The fluorescence properties of pyranine depend on its protonation state (Fig. 1c, Supplementary Fig. 4). After suitable calibration measurements (Supplementary Fig. 5), we could hence monitor the pH optically^29^. Figure 1d plots the fluorescence intensity ratio over time while the system was exposed to light-dark cycles (Supplementary Movie 1, Supplementary Fig. 6). Notably, we obtained the same results as previously with the pH electrode.

### Light-harvesting *E. coli* as internal pH actuators

**Fig. 2:**
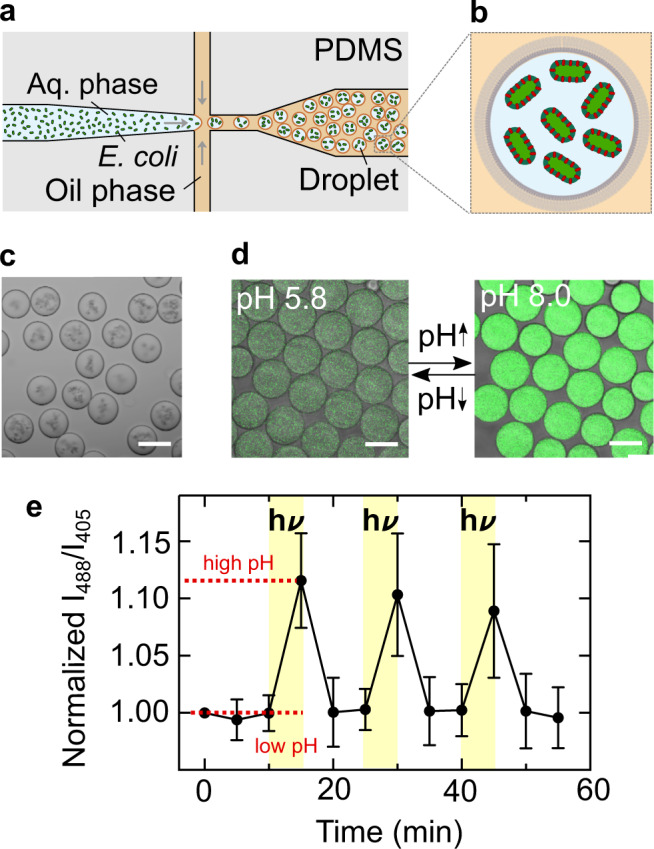
*E. coli* as light-activated synthetic organelles that change the pH inside cell-sized confinement. **a** Schematic illustration of the microfluidic device used to encapsulate engineered *E. coli* and pyranine into cell-sized compartments. Water-in-oil droplets were generated at a flow-focusing T-junction of a PDMS-based device. **b** Schematic illustration of a surfactant-stabilized water-in-oil droplet containing engineered *E. coli*. **c** Brightfield image of monodisperse water-in-oil droplets with a radius of 27 ± 5 μm (mean ± s.d., *n* = 53) containing engineered *E. coli* (OD_600_ = 20). Scale bar: 50 μm. **d** Overlay of confocal fluorescence and brightfield images of pyranine (c= 50 μM, *λ*_*ex*_ = 488 nm) inside droplet-based compartments at pH 5.8 and pH 8.0. Scale bar: 50 μm. **e** Normalized fluorescence intensity ratio I_488_/I_405_ of *E. coli* and pyranine-containing droplets over time. The fluorescence intensity ratio (mean ± s.d., *n* = 11 droplets) of pyranine (and hence the pH) increases reversibly during periods of illumination with white light (30 W halogen bulb, highlighted in yellow). Note that the number of recorded frames was reduced because the illumination light had to be turned off each time an image was acquired, which will bias the proton pumping activity. Source data is available for Fig. 2e.

Having demonstrated light-activated pH switching in bulk, we wanted to integrate the engineered *E. coli* as artificial mitochondria mimics in synthetic cell-sized confinements. Using a microfluidic droplet formation device (Fig. 2a), *E. coli* and pyranine were encapsulated in surfactant-stabilized water-in-oil droplets (Fig. 2b; Supplementary Fig. 7). We obtained *E. coli*-containing compartments with a radius of 27 ± 5 μm (mean ± s.d., n = 53, Fig. 2c). Pyranine served as a fluorescent pH indicator inside the compartments (Fig. 2d; Supplementary Fig. 8). We exposed the system to three consecutive light-dark cycles. Illumination with white light triggered a pH increase inside the cell-sized compartments due to the light-driven proton transport by the *E. coli*, resulting in an optical response of the compartments themselves (Fig. 2e; Supplementary Movie 2). Taken together, we demonstrated that the genetically engineered *E. coli* can provide light-activated proton gradients in cell-sized compartments.

### pH-sensitive attachment of DNA to the compartment periphery

Proton gradients in synthetic systems are especially exciting if they can be utilized to control and energize downstream processes. Instead of relying on purified proteins, an increasingly popular approach is to construct such pH-dependent machineries *de novo* from molecular building blocks. DNA nanotechnology, in particular, has been employed to build a variety of functional components for synthetic cells^17,30,31^, including membrane-sculpting^32–35^ and pH-responsive components such as filaments^36^ or rotors^37,38^. However, pH-responsive actuation is challenging after encapsulation into a compartment. With the *E. coli*, we can circumvent this by converting light into a proton gradient.

**Fig. 3:**
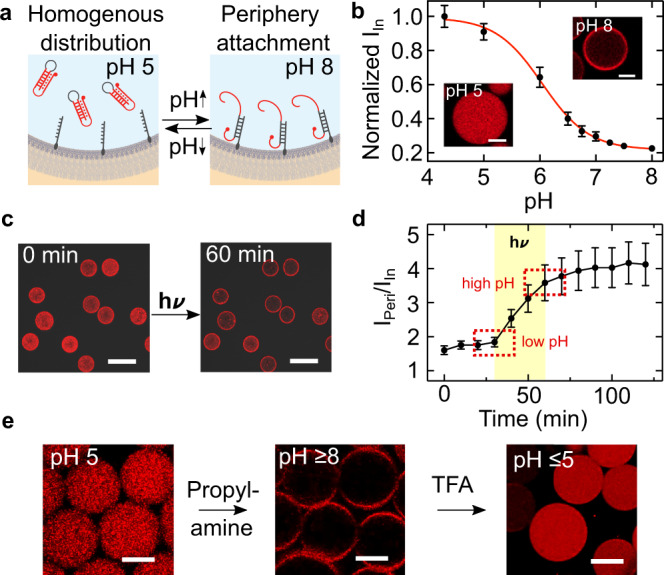
pH-sensitive DNA attachment to the droplet periphery stimulated with engineered *E. coli*. **a** Schematic illustration of pH-sensitive duplex formation at the droplet periphery. In response to higher pH, the DNA triplex motif opens up and reversibly attaches to the cholesterol-tagged DNA handles at the compartment periphery. **b** Normalized fluorescence intensity of triplex-forming DNA inside the droplet (excluding the periphery) dependent on the pH (mean ± s.d., *n* = 20). The sigmoidal fit (red curve) has a turning point at pH 6.05. The insets depict confocal fluorescence images of Cy5-labeled triplex-forming DNA (*λ*_*ex*_ = 633 nm, 1 μM) inside a water-in-oil droplet (containing 1.5 μM cholesterol-tagged DNA) at pH 5 (bottom left) and pH 8 (top right). At pH 8, the triplex-forming DNA is located at the droplet periphery, whereas it is homogeneously distributed at pH 5. Scale bars: 20 μm. **c** Confocal images of microfluidic water-in-oil droplets containing the triplex-forming DNA (*λ*_*ex*_ = 633 nm), cholesterol-tagged DNA and engineered *E. coli* before (0 min) and after (60 min) illumination with white light. Scale bars: 100 μm. **d** Fluorescence intensity ratio I_peri_/I_in_ (mean ± s.d., *n* = 20) of the triplex-forming DNA over time. The ratio increases during light illumination due to binding of the triplex-forming DNA to the droplet periphery. The time period of illumination is indicated in yellow. **e** Confocal images of microfluidic water-in-oil droplets containing the triplex-forming DNA (*λ*_*ex*_ = 633 nm) and cholesterol-tagged DNA produced at pH 5 (left image). Flushing of the proton acceptor propylamine (1 vol% in HFE) led to a pH increase of the aqueous solution inside the droplets and hence attachment of the triplex-forming DNA (middle). Subsequent flushing of the proton donor trifluoroacetic acid (1 vol% in HFE) decreased the pH and hence causes DNA detachment (right). The attachment of triplex-forming DNA to the droplet periphery is reversible. Scale bars: 30 μm. Source data is available for Fig. 3b, d.

Towards this goal, we want to implement pH-induced membrane modification and remodeling. For this purpose, we employ a single-stranded DNA sequence, which consists of specifically designed sections^36,39^: First, it contains a self-complementary section, which forms a DNA duplex following the Watson-Crick basepairing rules. A single-stranded hairpin loop connects the duplex-forming sections. Another critical single-stranded region is located at the 3ʹ end. At acidic pH it wraps around the DNA duplex to form a triplex, held together by Hoogsten interactions. At basic pH, the triplex becomes unstable. The remaining duplex can now also open up, if a second DNA strand with higher affinity binds to the hairpin loop^36^. By functionalizing this second DNA strand with a terminal cholesterol tag, it self-assembles at the compartment periphery due to hydrophobic interactions^40^. Thereby, we can recruit the triplex-motif strand to the compartment-periphery in a pH-reversible manner (Fig. 3a). At basic pH, the triplex-motif strand is bound to the periphery (Fig. 3b, inset top right and Supplementary Fig. 9). At acidic pH, on the other hand, it remains homogeneously distributed inside the compartment (Fig. 3b, inset bottom left). Note that the periphery attachment is due to specific interactions between the opened DNA triplex and the complementary cholesterol-tagged DNA. Unspecific absorption in the absence of the cholesterol-tagged DNA was not observed (Supplementary Fig. 10)^41^. To characterize the pH-sensitive membrane attachment, we assessed the fluorescence intensity inside the compartment as a function of pH. The fluorescence intensity decrease with increasing pH follows a sigmoidal fit with a pK_a_ of 6.05, which is compatible with the pH range of the *E. coli* and previous works^39^. It is important to note that the choice of fluorophore can affect the pH switching point^41^.

As a next step, we need to verify that membrane attachment of the DNA can also be triggered by the engineered *E. coli*. We hence co-encapsulated them with the cholesterol-tagged as well as the triplex-forming DNA strand using a microfluidic two-inlet device (Supplementary Figs. 7 and 11). A second inlet proved to be advantageous, because the cholesterol-tagged DNA could bind to the droplet periphery before encountering the *E. coli*, hence preventing unwanted attachment to the *E. coli* due to hydrophobic interactions^42^.

After microfluidic droplet formation in the dark, the triplex-forming DNA was homogeneously distributed inside the compartment with some attachment to the periphery (Fig. 3c). From the calibration curve, we could deduce a starting pH value of around 6.2 inside the droplets, consistent with previous experiments in Fig. 1. Upon illumination, the DNA attached to the compartment periphery over the course of 30 min (Fig. 3d, Supplementary Movie 3). We can deduce a pH increase of approximately one pH unit to about pH 7.25, consistent with the bulk experiments in Fig. 1 (Supplementary Note 1). The dynamic opening of the triplex and subsequent attachment to the periphery was considerably slower than the pyranine response^36^. We observed that the DNA remained attached to the compartment periphery after the light was turned off. We found that this is due to an interesting hysteresis effect: Once the DNA duplex at the droplet periphery was formed, the detachment of the triplex-forming DNA was shifted to substantially lower pH values (Supplementary Fig. 12). Therefore, the DNA did not detach when the pH returned to its original value after turning the light off. This effect can likely be attributed to an effective stabilization of the duplex conformation^41^. Detachment could, however, be achieved with larger pH gradients (Supplementary Movie 4): Fig. 3e shows the reversible attachment of the DNA triplex to the compartment periphery, triggered by the addition of a proton acceptor (1 vol% propylamine in HFE) and subsequent addition of a proton donor (1 vol% trifluoroacetic acid (TFA) in HFE). The increase in fluorescence after addition of TFA can be attributed to the pH-sensitive nature of the Cy5 dye^43^.

We have thus realized a complex reaction pathway, where illumination activates the internal organelle mimics, causing a proton gradient which, in turn, leads to the stable modification of the compartment periphery. Moreover, the pH-sensitive membrane attachment and the discovered hysteresis effect extend the scope of the DNA triplex motif in DNA nanotechnology.

### pH-induced morphology change

**Fig. 4:**
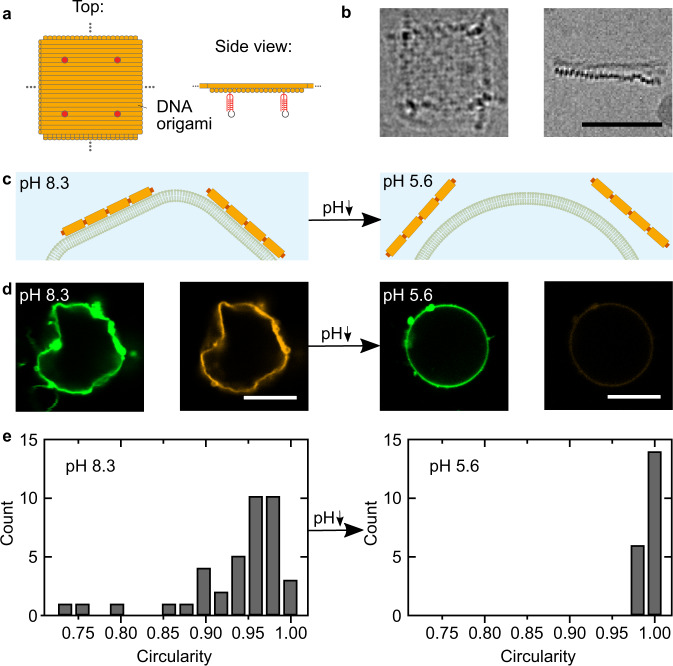
Deformation of GUVs with pH-sensitive DNA origami. **a** Schematic illustration of the DNA origami, which can polymerize into flat DNA origami sheets due to blunt end stacking. The DNA origami was functionalized with four DNA triplex motifs (red, two are shown), such that its assembly on the GUV membrane is pH-dependent. **b** Cryo-EM micrographs of the DNA origami plates. The top view (left) and the side view (right) showing the two DNA layers connected at a 90^∘^ angle. Scale bar: 50 nm. **c** Schematic illustration of a section of a GUV membrane functionalized with cholesterol-tagged pH-sensitive polymerized DNA origami. At high pH the DNA origami sculpts the GUV membrane. At low pH, it detaches and the GUV relaxes into its spherical shape. (Continued on the following page) **d** Confocal images of GUVs before (left) and after (right) decreasing the pH from pH 8.3 to pH 5.6 by addition of iso-osmotic potassium dihydrogenphosphate buffer. The GUV (lipids labeled with Atto488, *λ*_*ex*_ = 488 nm) is initially deformed due to the membrane-bound polymerized DNA origami (labeled with Cy3, *λ*_*ex*_ = 561 nm). The DNA origami detaches upon lowering the pH (the fluorescence from the detached DNA origami in the background is too weak to be visible). Scale bars: 10 μm. **e** Histograms of GUV circularity before (left) and after (right) lowering the pH. At pH 8.3, the mean circularity is 0.94 ± 0.06 (n = 39) compared to 0.991 ± 0.004 (*n*=20) at pH 5.6, respectively. Source data is available for Fig. 4e.

Next, we can exploit the pH-responsive modification of the compartment periphery to provide a meaningful function. Assuming that the DNA triplex motif could serve as a shuttle to bring components to the periphery, we set out to develop a cytoskeleton mimic, which could sculpt synthetic cellular compartments in a pH-responsive manner. For this purpose, we designed a DNA origami plate made of two layers of DNA helices (Fig. 4a, Supplementary Fig. 13). The two layers were twisted at a 90^∘^ angle as visible in the cryo electron micrographs (Fig. 4b), providing interaction sites for blunt-end stacking^44^ on all four sides of the DNA origami. This, in turn, leads to efficient polymerization of the DNA origami monomers into large flat sheets when the edge staples at the scaffold seam are included as verified via cryo-electron microscopy (Supplementary Fig. 14), atomic force microscopy (Supplementary Fig. 15) and agarose gel electrophoresis (Supplementary Fig. 16). The bottom-side of the DNA origami was functionalized with the DNA triplex motif at four positions. At basic pH, the DNA origami thus attached to the periphery of cell-sized droplets functionalized with the complementary cholesterol-tagged strand. However, the droplets remained spherical (Supplementary Fig. 17). This is not surprising given that droplets could also not be deformed with cytoskeletal proteins due to their interfacial properties^17,45^. We thus moved to a compartment system which better mimics the mechanical properties of cellular membranes. We produced giant unilamellar lipid vesicles (GUVs) and functionalized them externally with the cholesterol-tagged DNA. We then added the pH-sensitive DNA origami to the GUVs at pH 8.3. At this pH, the DNA origami binds to the GUV membrane. Upon addition of the staples at the scaffold seam, which enable blunt-end stacking, we observed considerable deviations from the initially spherical shape of the GUV (Fig. 4c, d). Large flat sections appeared on the GUV with kinks at the phase boundaries between the polymerized flat DNA sheets. In fluorescence recovery after photobleaching (FRAP) experiments, we find that the polymerized DNA origami layer is not diffusive, as expected for large interconnected sheets, in particular in the presence of Mg^2+^^46^. For the underlying deformed lipid membrane, we obtain a diffusion coefficient of 1.23 ± 0.14 μm^2^s^−1^ which is comparable to the lipid diffusion in bare GUVs^47^ (Supplementary Fig. 18). In addition to the morphological change, we observe a suppression of membrane fluctuations (Supplementary Fig. 19, Supplementary Movie 5), indicating a mechanical stabilization of the compartment^35,48^ by the DNA-based exoskeleton mimic (Supplementary Note 2). The stabilization effect could potentially be exploited for drug delivery applications. Both the morphological and the mechanical alterations are reversible (Supplementary Fig. 20): Addition of an acid led to pH decrease and hence to the detachment of the DNA origami from the GUV membrane. Notably, the GUVs relax back into their initial spherical shape (Fig. 4d, more images in Supplementary Figs. 21 and 22). Note that a pH decrease to pH 5.6 is required to fully detach the DNA origami from the GUV membrane, which is below the pH decrease that can be provided by the *E. coli* (see Fig. 1). The larger pH gradients required for attachment and detachment of the DNA origami compared to the triplex strand alone can be explained with a cooperativity effect. Each DNA origami is modified with four triplexes. Therefore, complete detachment took several hours and hence the addition of an acid was necessary. The histograms in Fig. 4e quantify the pH-reversible morphology change of the GUVs, revealing lower and more broadly distributed circularities when the DNA origami was attached at high pH. Taken together, the self-assembly of nanoscopic pH-responsive building blocks could trigger the microscopic morphological remodeling of the shape of lipid-membrane-based synthetic cellular compartments.

**Fig. 5:**
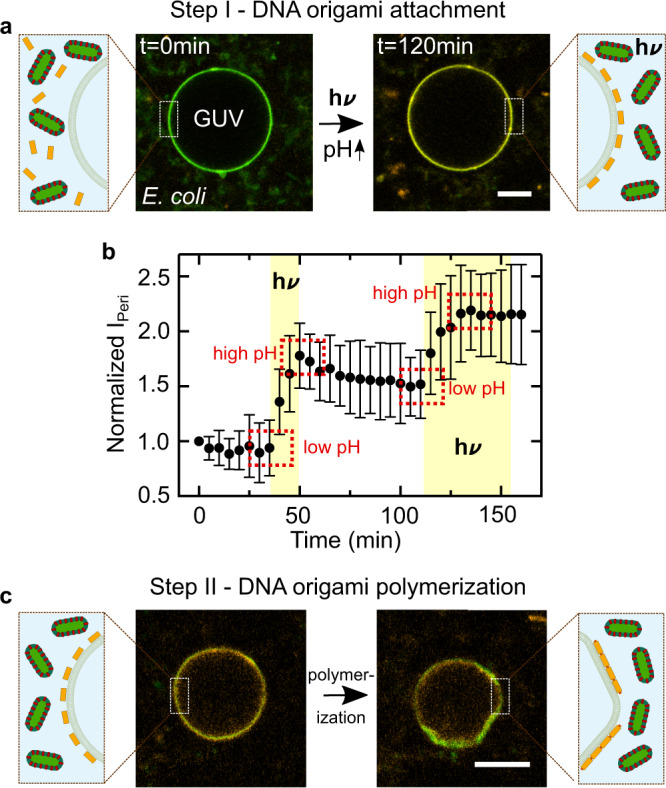
Light-harvesting *E. coli* trigger DNA origami attachment and GUV deformation. **a** Confocal images of a GUV (*λ*_*ex*_ = 488 nm, green) functionalized with cholesterol-tagged DNA immersed in an *E. coli* and DNA origami (*λ*_*ex*_ = 561 nm, orange) containing solution before (left) and after (right) light illumination. Scale bar: 10 μm. After light illumination, the DNA origami (orange) attaches to the GUV due to the pH increase triggered by the *E. coli*. **b** Normalized fluorescence intensity I_peri_ (mean ± s.d., *n* = 11) of the triplex-forming DNA at the GUV periphery monitored over time. The time period of illumination is indicated in yellow, illumination leads to a pH increase and hence DNA origami attachment. **c** Confocal images of a GUV (*λ*_*ex*_ = 488 nm, green) after light-mediated DNA origami (*λ*_*ex*_ = 561 nm, orange) attachment to the membrane and addition of the DNA staple strands at the scaffold seam which enable blunt-end stacking. DNA origami polymerization leads to the deformation of the GUV membrane within 2 h. Scale bar: 10 μm. Source data is available for Fig. 5b.

Finally, we set out to combine the DNA origami-mediated pH-sensitive deformation of GUVs with the light-responsive proton-pumping capabilities of the *E. coli*. First, we showed that the GUVs remained stable in the *E. coli* culture and that we can attach the plain triplex-forming DNA to the GUV membrane upon illumination (Supplementary Figs. 23 and 24; Supplementary Movie 6). Thus, the pH-signal-transduction between the top-down engineered *E. coli* and bottom-up assembled synthetic cells is also successful when the *E. coli* are used as external actuators. Next, we immersed the GUVs in a solution of *E. coli* and pH-sensitive DNA origami. We observed the attachment of the pH-sensitive DNA origami to the GUV membrane upon illumination (Fig. 5a, Supplementary Movie 7). Fig. 5b quantifies the amount of DNA origami attachment, i.e., normalized fluorescence intensity at the GUV periphery *I*_Peri_, over time during periods of illumination and darkness. DNA origami attachment happens repeatedly and only during periods of illumination, until saturation is reached. The attachment occurs due to the pH increase triggered by the light-harvesting *E. coli*. After illumination, in periods of darkness, the pH gradient dissipates. Nevertheless, the amount of DNA origami attachment remains roughly constant (Fig. 5b, *t* = 50 min until *t* = 110 min). This can be attributed to the observed pH hysteresis effect (Supplementary Fig. 12): The pH would have to drop below the starting value for detachment, which cannot be achieved with the *E. coli* alone. Nevertheless, a second illumination cycle (from *t* = 110 min) showed that the *E. coli* remain active and that the DNA origami attachment continued until saturation was reached.

After attachment, we enabled the polymerization of individual DNA origami monomers by adding the staple strands at the scaffold seam, which induce blunt-end stacking. This leads to the deformation of GUVs within two hours (Fig. 5c, Supplementary Movie 8), when the solution was illuminated with light, whereas GUVs remained spherical when they were left in the dark (Supplementary Figs. 25 and 26). Note that the deformation is weaker compared to the deformation achieved with conventional pH switching due to the smaller pH gradient. We can thus exploit the light-harvesting *E. coli* to actuate a morphology change of the GUVs.

## Discussion

In summary, we have shown that the use of top-down engineered bacteria can enhance bottom-up assembled synthetic cells. The light-induced proton gradients we achieve with xenorhodopsin-overexpressing *E. coli* are not only larger than what was previously achieved with purified and reconstituted proteins – we also circumvent the laborious processes involved in their preparation. Especially membrane proteins, which can provide transient or chemically storable forms of energy as well as signal transduction and molecular transport in living cells, can be challenging to purify and reconstitute. Therefore, we can exploit the engineered *E. coli* to drive sophisticated downstream dynamics in synthetic cells. In particular, we demonstrate the pH-sensitive attachment of a triplex-motif-carrying DNA origami to the compartment periphery upon illumination. The polymerized DNA origami, in turn, leads to a shape change of the GUVs triggered by the proton-pumping activity of the *E. coli*. The possibility to manipulate lipid membranes and not just the DNA nanostructures themselves broadens the scope of the popular DNA triplex-motif. For biotechnological applications, compartments that modify themselves as a response to environmental factors are highly desirable. More general, the integration of top-down engineered cells into bottom-up synthetic biology, contributing to bridge a long-standing divide, will provide the potential to realize diverse functions beyond light-harvesting^49^. We envision that the integration of top-down engineered components in synthetic cells will be a leap forward in their complexity and functionality.

## Methods

### Cloning

The plasmid pNR31 harboring the xenorhodopsin gene from Nanosalina (NsXeR) fused to the gene coding for superfolder-GFP (sf-GFP) was assembled by replacing the gene coding for proteorhodopsin in plasmid pNR03^7^ with the NsXeR gene (Supplementary Table 1). Therefore, a codon-optimized NsXeR gene based on the amino-acid sequence^25^ with a 5ʹ NdeI and a 3ʹ BamHI restriction site was synthesized by GenScript (https://www.genscript.com) and cloned into the pUC57 plasmid. Using these two restriction enzymes (New England Biolabs, Ipswich, MA), the NsXeR gene was then subcloned into the pNR03 plasmid. The plasmid pNR33 harboring the NsXeR gene fused to mCherry (Supplementary Table 1) was assembled in multiple steps. First the sf-GFP gene in pNR03 was replaced by the gene coding for mCherry. To that end, the mCherry gene was amplified from the pNR09 plasmid using primers 5ʹ-GGC GGA TCC ATG CAT AGC AAG GGC GAG-3ʹ and 5ʹ-GCC AAG CTT CTT GTA CAG C-3ʹ (Microsynth AG) to introduce 5ʹ BamHI and 3ʹ HindIII restriction sites^7^. The resulting PCR-product was then cloned into plasmid pNR03 where it replaced the sf-GFP gene. Subsequently the same subcloning as for plasmid pNR31 was performed to replace the gene coding for proteorhodopsin with the NsXeR gene.

### Overexpression of fusion-proteins in *E. coli*

*E. coli* C41 (DE3) cells (Sigma-Aldrich) were transformed with the plasmids pNR31 and pNR33. 100 mL Luria-Bertani (Fisher Scientific) liquid cultures (100 μg/mL ampicillin, Sigma-Aldrich) were inoculated 1:100 from overnight cultures. The *E. coli* cells were grown at 37^∘^C while shaking at 220 rpm until an OD_600_ of 0.4 was reached. Then, all-trans-retinal (Sigma-Aldrich) was added to a concentration of 10 μM and the expression of the fusion-proteins was induced with the addition of 1 mM isopropyl-D-thiogalactopyranoside (IPTG, Sigma-Aldrich). The cells were incubated for another 4 h at 37 ^∘^C while shaking at 220 rpm. Subsequently they were harvested by centrifugation (3200 × *g* for 10 min at 4 ^∘^C) and resuspended in 150 mM NaCl. The cells were stored at 4^∘^C and protected from light until further use.

### Photoactivity measurements with a micro pH-electrode

*E. coli* cells overexpressing either XeR-GFP or XeR-mCherry were washed twice with 150 mM NaCl (3200 × g for 10 min at 4 ^∘^C) prior to photoactivity measurements. Immediately before the measurement, another washing step was performed. The bacteria were concentrated to an OD_600_ of 20. Photoactivity measurements were conducted using a micro pH-electrode (InLab Micro Pro, Mettler Toledo, Columbus, OH) and a sample volume of 800 *μ*L. The pH was recorded every 10 s. During the measurements the bacteria were protected from ambient light and continuously stirred to prevent sedimentation. The sample was illuminated with a KL 1500 LCD halogen lamp (Schott) for 5 min during each light-dark cycle. After each illumination-period the sample was kept in the dark for 10 min. All measurements were performed at room temperature.

### Confocal fluorescence microscopy

A confocal laser scanning microscope LSM 880, LSM 800 or LSM 700 (Carl Zeiss AG) was used for confocal imaging. The pinhole aperture was set to one Airy Unit and experiments were performed at room temperature. The images were acquired using a 20x objective (Plan-Apochromat 20x/0.8 M27, Carl Zeiss AG). Images were analyzed and processed with ImageJ (NIH, brightness and contrast adjusted) and Zen imaging software (Version 2.3). Confocal images were analyzed using a custom-written ImageJ macro (available here: 10.5281/zenodo.4738934). Cell-sized compartments were identified, their radius calculated and the intensity within the compartment center defined as mean inner intensity I_In_. The peripheral intensity was determined by quantifying the maximum intensity along a line orthogonal to the compartment periphery. This was repeated every 18^∘^ and the mean value taken as I_Peri_. The resulting data was plotted with Prism 8 (Version 8.4.3) and figures were compiled with Inkscape (Version 1.0rc1).

### Formation of surfactant-stabilized water-in-oil droplets

Microfluidic PDMS-based (Sylgard 184, Dow Corning) devices for the formation of water-in-oil droplets were produced and assembled^40^. The device layouts of the single and two-inlet devices are shown in the Supplementary Fig. 7. The oil-phase contained 1.4 wt% of Perflouro-polyether-polyethylene glycol (PFPE-PEG) block-copolymer fluorosurfactants (PEG-based fluorosurfactant, Ran Biotechnologies, Inc.) dissolved in HFE-7500 oil (DuPont). The aqueous phase contained the encapsulated content and was varied as described in the corresponding sections. The fluid pressures to induce droplet formation were controlled by an Elveflow microfluidic flow control system or syringe pumps (Harvard Apparatus). The fluids for the syringe pumps were injected into the channels with 1 ml syringes (Omnifix, B.Braun, Germany) connected by a cannula (Sterican®0.4 ×20 mm, BL/LB, B.Braun) as well as PTFE-tubing (0.4 × 0.9 mm, Bola). To observe the droplet production process, an Axio Vert.A1 (Carl Zeiss AG) inverse microscope was used. As an alternative to the microfluidic formation of droplets, the aqueous phase was layered on top of the oil phase within a microtube (Eppendorf) and droplet formation was induced by manual shaking^50^.

### Photoactivity measurements in droplets

Photoactivity measurements in droplets were performed by encapsulating *E. coli* (OD_600_ ≈ 20) with pyranine (50 μM) into surfactant-stabilized droplets using the microfluidic device described above. The droplets were stored at 4^∘^C after formation to allow for equilibration of the pH inside the droplet. Subsequently, droplets were sealed in an observation chamber and observed with confocal fluorescence microscopy. After 10 min of imaging in the dark, the sample was illuminated for 5 min using a Photonic PL 1000 lamp (light intensity 8 Mlx using a 30 W halogen bulb). The lightguide was placed 5-10 cm above the sample. These cycles were repeated for 1 h.

### pH-sensitive attachment of DNA to the droplet periphery

Cholesterol-tagged DNA (sequence: 5ʹ (Cy3)-ACCAGACAATACCACACAATTTT-CholTEG 3ʹ, HPLC purified) and the Cy-5 labeled triplex-forming DNA (sequence: 5ʹ Cy5-TTCTCTTCTCGTTTGCTCTTCTCTTGTGTGGTATTGTCTAAGAGAAGAG 3ʹ, adapted from Green et al.^36^, HPLC purified) were purchased from Biomers or Integrated DNA Technologies. Both DNA sequences were encapsulated in microfluidic droplets at a concentration of 1.5 μM and 1 μM, respectively. For the calibration measurement (Fig. 3b), the aqueous solution inside the droplets additionally contained 50 mM potassium phosphate buffer at the respective pH. Propylamine (from Sigma Aldrich) and Trifluoracetic Acid (TFA, from Sigma Aldrich) were flushed to dynamically change the pH of the droplets’ aqueous phase. For the co-encapsulation of the DNA together with the *E. coli* (OD_600_ ≈ 20), a two-inlet droplet formation device was used (see Supplementary Fig. 7). Droplets were sealed in an observation chamber for confocal fluorescence imaging experiments.

### GUVs electroformation and DNA attachment

GUVs consisting of 99 % DOPC (1,2-dioleoyl-sn-glycero-3-phosphocholine, from Avanti Polar Lipids) and 1 % Atto488-DOPE (1,2-dioleoyl-sn-glycero-3-phosphoethanolamine-Atto488, from AttoTEC) in 120 mM sucrose were produced via electroformation using a Vesicle Prep Pro (Nanion)^34^. An AC-current with an amplitude of 3 V and a frequency of 5 Hz was applied for 2 h at 37^∘^C. The cholesterol-tagged DNA and the triplex-forming DNA were added to the GUVs at a concentration of 0.6 μM and 0.4 μM, respectively, before the addition of the *E. coli* (OD_600_ ≈ 20), in an unbuffered solution containing 150 mM NaCl and 5 mM MgCl_2_.

### DNA origami design and assembly

DNA origami structures were adapted from an earlier design by Kopperger et al.^51^ using the open-access software *cadnano*^52^. Several changes were introduced, in particular: (1) Addition of nine DNA staple strand overhangs on the top layer, complementary to single stranded fluorescent Cy3-tagged DNA; (2) Addition of four single stranded overhangs on the bottom layer, complementary to the triplex-forming DNA; (3) Complete redesign of the edge staples resulting in a cross-shaped plate. The sticky cross DNA origami contained edge staples that finish the scaffold seam, enabling blunt-end stacking with neighboring origami. (4) Use of the longer single-stranded scaffold DNA, type p8064. A complete list of the DNA sequences is shown in Supplementary Data 1, the details of the design are shown in Supplementary Fig. 13. DNA origami was assembled by adding all unmodified staple strands (Integrated DNA Technologies, Inc., purification: standard desalting) in fivefold excess compared to the p8064 scaffold strand (tilibit nanosystems GmbH). The solution contained 1 × TAE (Tris-Acetate-EDTA, Sigma-Aldrich) and 20 mM MgCl_2_ (Sigma-Aldrich) at pH 7.4. The DNA origami was annealed in a thermocycler (Bio-Rad T100) that controls a temperature ramp from 70 ^∘^C to 20 ^∘^C over 12 h and successively holds the temperature at 40 ^∘^C for at least 3 h^51^. The unpurified samples were stored at 4 ^∘^C until further use.

### Purification of the DNA origami

Prior to purification from excess staples, the DNA origami was mixed with 1 μM Cy3-tagged single-stranded DNA (Integrated DNA Technologies, Inc., DNA sequence: 5ʹ Cy3- AAAAAAAAAAAAAAAAAAAA 3ʹ, purification: HPLC) as well as a pH-sensitive triplex-forming DNA motif (Integrated DNA Technologies, Inc., DNA sequence: 5ʹ TTCTCTTCTCGTTTGCTCTTCTCTTGTGTGGTATTGTCTAAGAGAAGAGTTTGATGCATAGAAGG 3ʹ). The DNA origami was then suspendend in 500 μL of 1 × TAE, 5 mM MgCl_2_ and purification was performed by spin filtration in a Biofuge Fresco microlitre centrifuge (Heraeus 75005521) using 100 kDa cutoff filters from Amicon (Amicon Ultra-15, PLHK Ultracel-PL Membran, UFC910008)^31^. After filtration, the MgCl_2_ concentration was raised to 20 mM again. Before the experiment, microvolume spectrophotometry (PEQLAB Biotechnologie GmbH) yielded a DNA origami concentration of 6.54 ± 0.42 nM.

### Cryo electron microscopy

In total, 3 μL of the assembled DNA origami in 10 mM sodium phosphate pH 8.3 containing 20 mM MgCl_2_ were blotted for 5-10 s in a (Vitrobot Mark IV, Thermo Fischer) on Quantifoli 2/1 grids with zero blot force at 100% humidity. Plunge frozen samples were imaged in a Krios equipped with a K3 camera behind an energy filter at a pixel size of 0.137 nm. Images were taken by single particle program (EPU, Thermo Fischer) with a a total dose of 20 e/A2. Movies of 20 frames were corrected^53^ then cropped, normalized, low-pass filtered (0.0625) and 4x binned^54^.

### GUV deformation with pH-sensitive DNA origami

The DNA origami (in 1 x TAE, 20 mM MgCl_2_) was incubated with cholesterol-tagged DNA at 50 nM for 25 minutes and immediately mixed with Atto488-labeled iso-osomotic (120 mOsmol) GUVs in a ratio of one to three. DNA origami-coated GUVs were imaged after 24 hours of incubation in the fridge. Subsequently, the GUVs were incubated for another 24 hours with 48 mM KH_2_PO_4_ buffer in order to detach the DNA origami from the GUV membrane.

### GUV deformation with pH-sensitive DNA origami and light-responsive *E. coli*

For these experiments, the DNA origami was suspended in a solution of 75 mM NaCl and 10 mM MgCl_2_ without addition of a pH-buffering agent. In order to prevent DNA origami polymerization prior to GUV-attachment, the staple strands at the scaffold seam were excluded. The single-stranded scaffold loops prevent base stacking. Subsequently, Atto488-labeled GUVs in sucrose (195 mOsmol) were diluted in 75 mM NaCl and 10 mM MgCl_2_ and mixed with 2 μM cholesterol-tagged DNA. After 5 min incubation, *E. coli* resuspended at an OD_600_ = 60 in 75 mM NaCl and 10 mM MgCl_2_ and DNA origami were mixed in a 1:1:1 ratio with GUVs. For the DNA origami attachment the solution was put into an observation chamber and illuminated with white light during confocal imaging. For subsequent GUV deformation, the staples at the scaffold seam were added to induce base stacking interactions between the membrane-bound DNA origami. The solution was illuminated for 30 min in bulk before the addition of 50 nM staple strands and imaged 14 h later.

### Statistics and reproducibility

The experiments were performed independently at least two times. In particular, values in Fig. 1b correspond to three independent experiments and in Fig. 1d to four independent experiments. Experiments for Figs. 2e, 3b, d and 4e were performed two times or more. Fig. 5 was only replicated once, however due to the sequential attachment via light and appropriate controls, we believe that this is adequate. All representative confocal, atomic force and electron microscopy images are only a subset of at least 10 or more images showing similar results.

## Supplementary information

Supplementary Information

Description of Additional Supplementary Files

Supplementary Movie 1

Supplementary Movie 2

Supplementary Movie 3

Supplementary Movie 4

Supplementary Movie 5

Supplementary Movie 6

Supplementary Movie 7

Supplementary Movie 8

Supplementary Dataset 1

## Data Availability

The data that support the findings of this study are available from the corresponding author upon reasonable request. Source data for main figures 1b, c, d, 2e, 3b, d, 4e, 5b and supplementary figures 1a, c, d, 2, 3a, b, 5, 8, 12, 16, 18b, 19a, b and 24c are provided with this paper.

## Source data

Source Data

## Code availability

Image J macro for the analysis of the intensity inside the compartment and at the compartment periphery is provided under the following link: 10.5281/zenodo.4738934.
