# Supplementary material for: Proton gradients from light-harvesting E. coli control DNA assemblies for synthetic cells
Source: Nat Commun. 2021 Jun 25;12:3967. doi: 10.1038/s41467-021-24103-x (PMC8233306; doi:10.1038/s41467-021-24103-x)
Supplement: Supplementary file 1 — Supplementary Information [file 41467_2021_24103_MOESM1_ESM.pdf]

Supplementary Information: Proton gradients from light-harvesting E. coli control DNA assemblies for synthetic cells

Kevin Jahnke,[†,‡] Noah Ritzmann,[¶] Julius Fichtler,[†] Anna Nitschke,[§] Yannik Dreher,[†,‡] Tobias Abele,[†,‡] Götz Hofhaus,[‖] Ilia Platzman,[§,⊥] Rasmus Schröder,[‖] Daniel J. Müller,[¶] Joachim P. Spatz,[§,⊥,#] and Kerstin Göpfrich[*,†,‡]

†*Max Planck Institute for Medical Research, Biophysical Engineering Group, Jahnstraße 29, 69120 Heidelberg, Germany*

‡*Department of Physics and Astronomy, Heidelberg University, 69120 Heidelberg, Germany*

¶*Department of Biosystems Science and Engineering, Eidgenössische Technische Hochschule (ETH) Zurich, Mattenstraße 26, 4058 Basel, Switzerland*

§*Max Planck Institute for Medical Research, Department of Cellular Biophysics, Jahnstraße 29, 69120 Heidelberg, Germany*

‖*Centre for Advanced Materials, Im Neuenheimer Feld 267, 69120 Heidelberg, Germany*

⊥*Institute for Molecular Systems Engineering (IMSE), Heidelberg University, Im Neuenheimer Feld 225, 69120 Heidelberg, Germany*

#*Max Planck School Matter to Life, Jahnstraße 29, 69120 Heidelberg, Germany*

E-mail: kerstin.goepfrich@mr.mpg.de

Contents

Supplementary Figures 4

Supplementary Figure 1: Activation of xenorhodopsin with different light sources. 4

Supplementary Figure 2: Saturation of pH-gradients upon white light illumination 5

Supplementary Figure 3: Photoactivity measurements with different *E. coli* densities . . . 6

Supplementary Figure 4: Chemical structure of pyranine . . . 7

Supplementary Figure 5: Calibration curve of pyranine fluorescence in presence of GUVs . . . 8

Supplementary Figure 6: Monitoring pH changes with pyranine in the presence of GUVs . . . 9

Supplementary Figure 7: Layout of the microfluidic device for the formation of water-in-oil droplets . . . 10

Supplementary Figure 8: Calibration curve of pyranine fluorescence in water-in-oil droplets . . . 11

Supplementary Figure 9: pH-sensitive attachment of triplex-forming DNA . . . 12

Supplementary Figure 10: Fluorophore-tagged single-stranded DNA does not interact with droplet-stabilizing surfactants . . . 13

Supplementary Figure 11: Brightfield and confocal images of microfluidic droplets containing engineered *E. coli* . . . 14

Supplementary Figure 12: Hysteresis of DNA triplex attachment and detachment 15

Supplementary Figure 13: Cadnano design of the membrane-sculpting DNA origami 16

Supplementary Figure 14: Blunt-end stacking induces polymerization of DNA origami plates . . . 17

Supplementary Figure 15: Atomic force microscopy images of the DNA origami . 18

Supplementary Figure 16: Agarose gel electrophoresis of the DNA origami . . . 19

Supplementary Figure 17: Droplets are not deformed by attaching DNA origami to the droplet periphery . . . 20
Supplementary Figure 18: FRAP experiments of GUVs with membrane-bound DNA origami . . . 21
Supplementary Figure 19: DNA origami cortex suppresses membrane fluctuations 22
Supplementary Figure 20: Deformation process of GUVs with DNA origami . . . 23
Supplementary Figure 21: Confocal images of deformed GUVs . . . 24
Supplementary Figure 22: Confocal images of GUVs after detachment of membrane-bound DNA-origami . . . 25
Supplementary Figure 23: Exemplary confocal images of DNA-functionalized GUVs 26
Supplementary Figure 24: Attachment of the single-stranded DNA triplex to GUVs during light illumination . . . 27
Supplementary Figure 25: Confocal images of deformed GUVs after light-mediated attachment of DNA origami . . . 28
Supplementary Figure 26: Light-mediated deformation of GUVs . . . 29

Supplementary Tables **30**

Supplementary Table 1: DNA and amino-acid sequences of xenorhodopsin-constructs . . . 30

Supplementary Notes **32**

Supplementary Note 1: Estimation of the pH change for DNA attachment . . . 32
Supplementary Note 2: Estimation of DNA origami density per GUV . . . 33

References **34**

Supplementary Figures

Supplementary Figure 1: Activation of xenorhodopsin with different light sources.

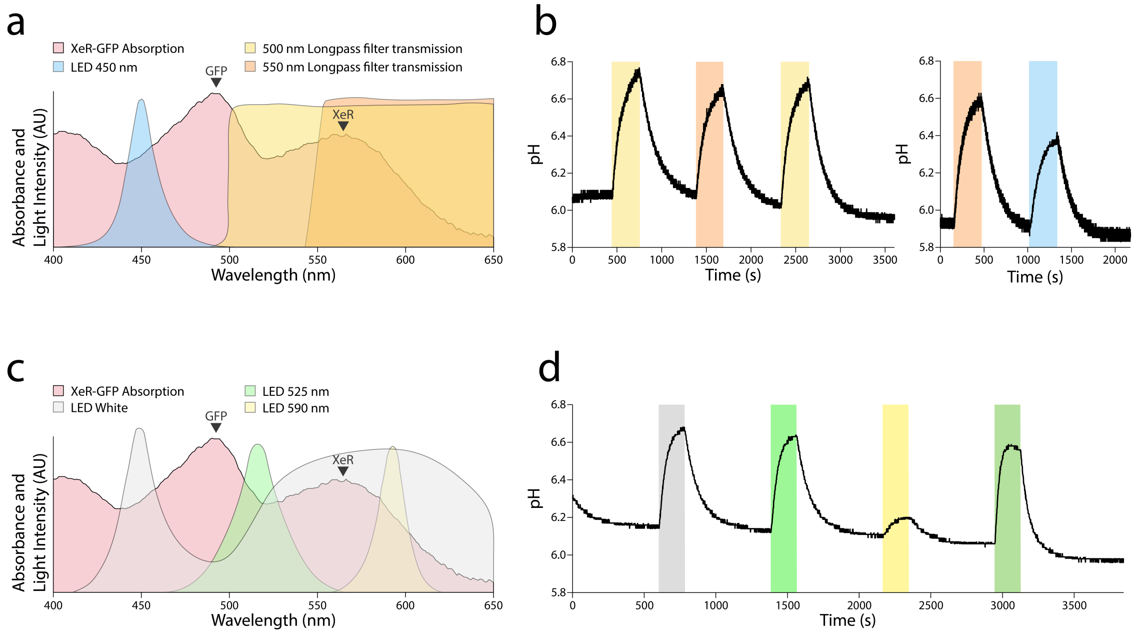

Figure 1: Activation of xenorhodopsin (XeR) with different light sources. **a** Absorption spectrum of the xenorhodopsin-GFP (XeR-GFP) fusion protein shown in light red. Respective absorption peak positions of xenorhodopsin and GFP are indicated. Relative light intensity and transmission profiles of filters and light sources are also shown. **b** Bulk photoactivity traces induced with the light sources presented in **a**. **c** Light intensity profiles of white, green and yellow LEDs plotted together with the XeR-GFP absorption spectrum. **d** Bulk photoactivity traces induced with the light sources shown in **c**. The dark green shaded region represents a combination of all three light sources shown in **c**.

Supplementary Figure 2: Saturation of pH-gradients upon white light illumination

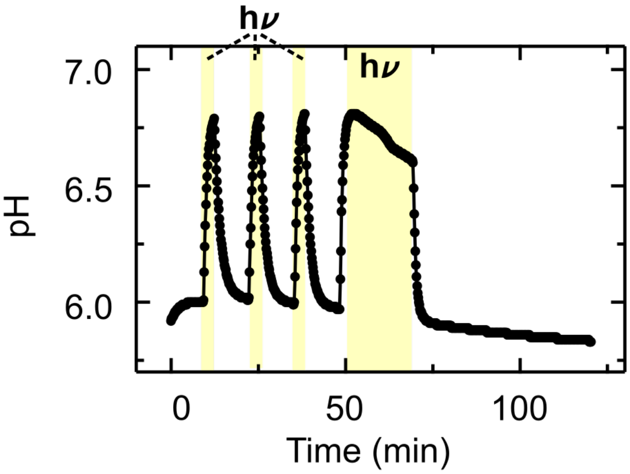

Figure 2: Engineered xenorhodopsin-expressing *E. coli* generate a pH-gradient upon illumination with white light, which saturates after ∼5 min of illumination. Photoactivity measurements of engineered *E. coli* (at OD_{600}=20, in 150 mM NaCl) with a pH-electrode. The pH is plotted as a function of time during four light-dark cycles (periods of illumination are indicated in yellow). The pH increases by ∼0.8 within 5 min of illumination and nearly returns to its original value after 10 min in the dark. A longer illumination time of 20 min (see final illumination period starting at $t = 50$ min) shows saturation of the pH-gradient and a slow decrease of the pH during continuous illumination.

Supplementary Figure 3: Photoactivity measurements with different *E. coli* densities

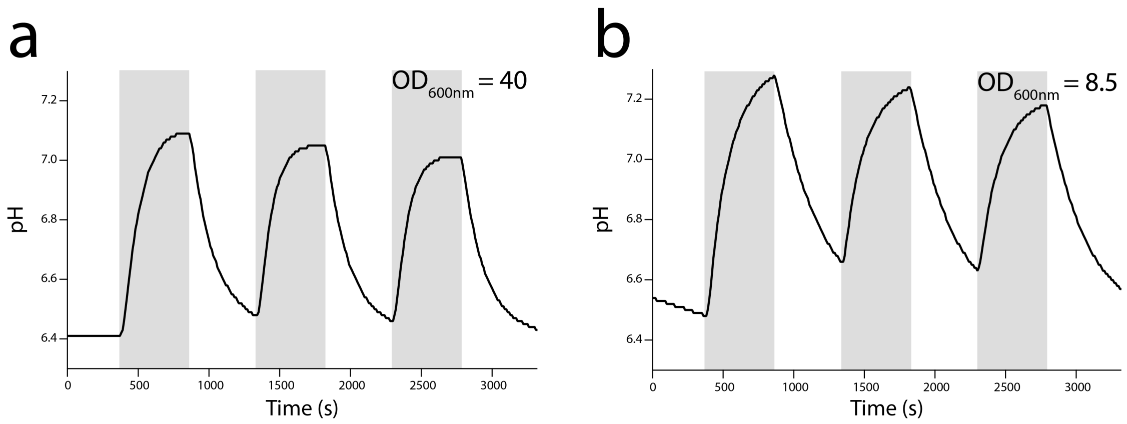

Figure 3: Photoactivity measurements with different E. coli densities. **a** Bulk photoactivity measurements with xenorhodopsin-GFP expressing *E. coli* at an OD_{600} of 40. **b** Bulk photoactivity measurements with the same XeR-GFP expressing *E. coli* as shown in **a**, diluted to an OD_{600} of 8.5. Illumination in both experiments was performed with a Schott, 1500 LCD lamp. The obtained pH gradients are very similar at both ODs, the kinetics are slightly increased at higher ODs. This could be explained by the hypothesis that the *E. coli* membrane itself cannot maintain pH gradients above one pH unit. Alternatively, it is conceivable that the light is partially absorbed by the denser *E. coli* solution.

Supplementary Figure 4: Chemical structure of pyranine

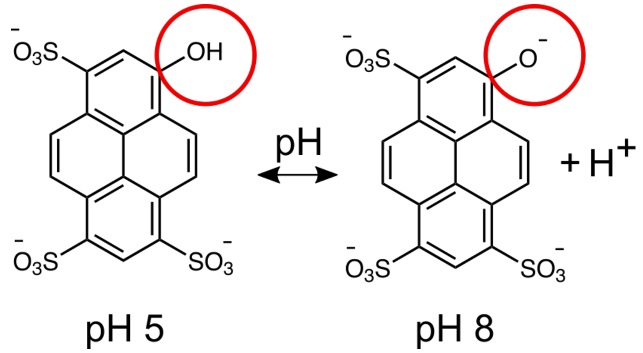

Figure 4: Chemical structure of pyranine at different pH values. The hydroxyl group of pyranine (indicated with a red circle) is deprotonated at high pH values leading to a change of the molecule's fluorescent properties (see Figure 1c). Therefore, pyranine can be used as pH-indicator by measuring the ratio of fluorescence emission upon excitation with the wavelengths 488 nm and 405 nm. A high ratio I_{488}/I_{405} indicates high pH values and low ratios a low pH value, respectively.

Supplementary Figure 5: Calibration curve of pyranine fluorescence in presence of GUVs

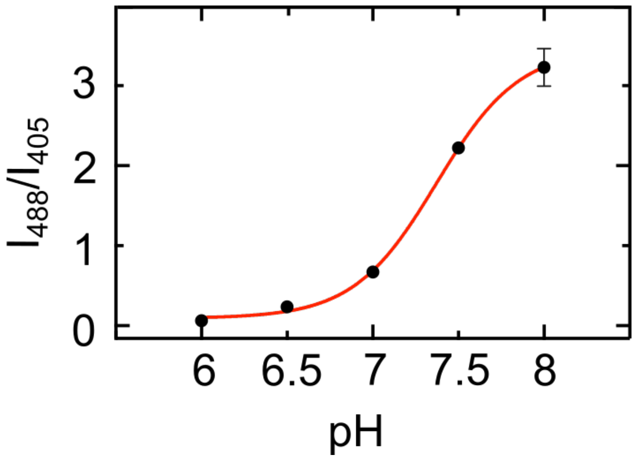

Figure 5: Calibration curve of pyranine fluorescence intensity (I_{488}/I_{405}) as a function of pH in a GUV-containing solution (150 mM sucrose, 50 µM pyranine, 5 mM $MgCl_2$, 100 mM sodium phosphate buffer pre-adjusted to the respective pH value). A sigmoidal fit (red) shows a pK_a value of pH 7.37. This is in very good agreement with the calibration performed in droplet-based compartments (see Supplementary Figure 8) and literature values.[1] Note that the pyranine fluorescence is strongly dependent on the buffer conditions. Therefore, the absolute values cannot be compared to the measurements in an *E. coli*-containing solution.

Supplementary Figure 6: Monitoring pH changes with pyranine in the presence of GUVs

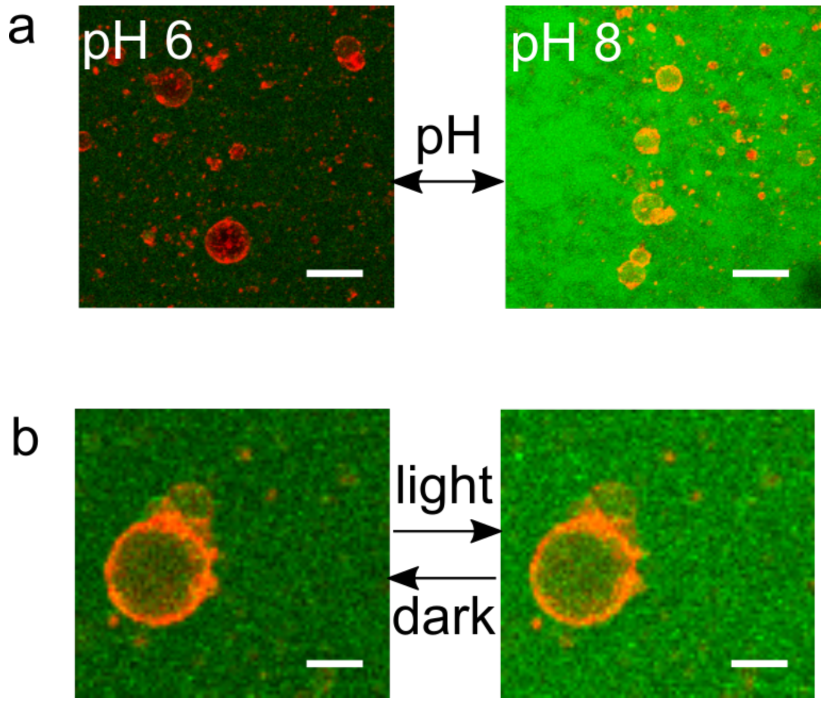

Figure 6: Monitoring pH changes with pyranine in the presence of GUVs. **a** Confocal fluorescence images of GUVs (red, λ_{ex} =647 nm) and pyranine (green, λ_{ex} =488 nm) at pH 6 and pH 8. The pyranine intensity upon 488 nm excitation increases with increasing pH. Scale bar: 50 µm. **b** Confocal fluorescence images of GUVs (red, λ_{ex} =647 nm) and pyranine (green, λ_{ex} =488 nm) in presence of engineered *E. coli* before (left) and after (right) white light illumination. White light illumination leads to an increase of the pH due to the proton-pumping activity of the *E. coli*, which can be visualized by an increase in pyranine fluorescence emission. Scale bar: 20 µm.

Supplementary Figure 7: Layout of the microfluidic device for the formation of water-in-oil droplets

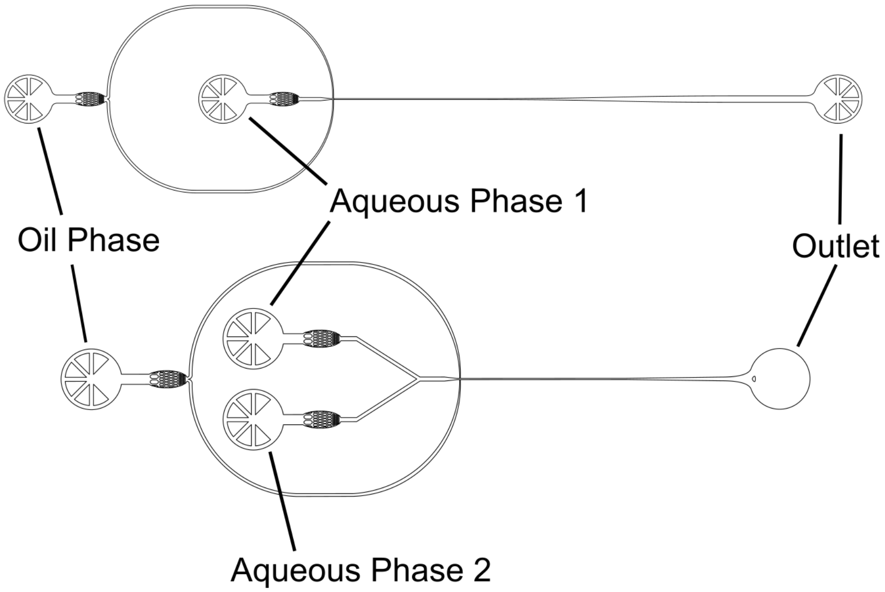

Figure 7: Layouts of single- (top) and two-inlet (bottom) microfluidic devices for the co-encapsulation of DNA and *E. coli* into surfactant-stabilized water-in-oil droplets. The cholesterol-tagged and triplex-forming DNA were supplied via one inlet and the *E. coli* via the second one to avoid attachment of the cholesterol-tagged DNA to the *E. coli* prior to droplet formation. The microfluidic PDMS devices (Sylgard184, Dow Corning, USA) were fabricated according to a previously published protocol [2] (see Methods). For confocal fluorescence imaging, the droplets were collected from the outlet and sealed in a simple observation chamber as described previously. [3]

Supplementary Figure 8: Calibration curve of pyranine fluorescence in water-in-oil droplets

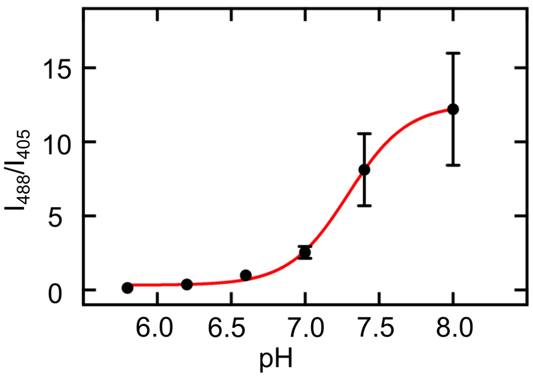

Figure 8: Calibration curve of pyranine fluorescence intensity I_{488}/I_{405} as a function of pH within water-in-oil droplets (50 µM pyranine, 5 mM $MgCl_2$, 50 mM sodium phosphate buffer pre-adjusted to the respective pH value). A sigmoidal fit (red) has a pK_a value of 7.29. This is in very good agreement with the calibration performed in presence of GUVs (see Supplementary Information Figure 5) and literature values.[1] Note that the pyranine fluorescence is strongly dependent on the buffer conditions. Therefore, the absolute values cannot be compared quantitatively to the measurements in *E. coli*-containing droplets. The data depicts mean values and error bars correspond to the standard deviation of n=22 droplets for pH 5.8, n=31 for pH 6.2, n=25 for pH 6.6, n=28 for pH 7.0, n=23 for pH 7.4 and n=24 for pH 8.0.

Supplementary Figure 9: pH-sensitive attachment of triplex-forming DNA

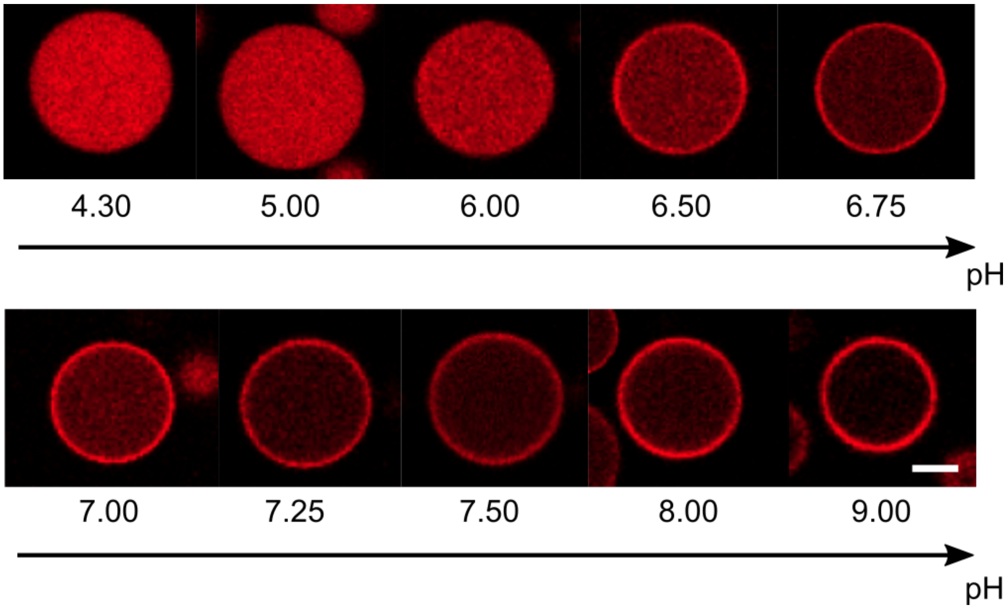

Figure 9: pH-sensitive attachment of triplex-forming DNA. Representative confocal fluorescence images of 1 µM Cy5-labeled triplex-forming DNA (red, λ_{ex} =647 nm) in droplet-based compartments at different pH values as indicated. The droplet periphery was functionalized with 1.5 µM cholesterol-tagged DNA (complementary to the hairpin region of the triplex, for DNA sequences see Materials and Methods). With increasing pH, Hoogsten interactions become weaker and an increasing amount of the triplex-forming DNA binds to the droplet periphery. For a quantitative plot of the fluorescence intensity inside the droplet a the different pH values, see Figure 3b (main text). Scale bar: 20 µm.

Supplementary Figure 10: Fluorophore-tagged single-stranded DNA does not interact with droplet-stabilizing surfactants

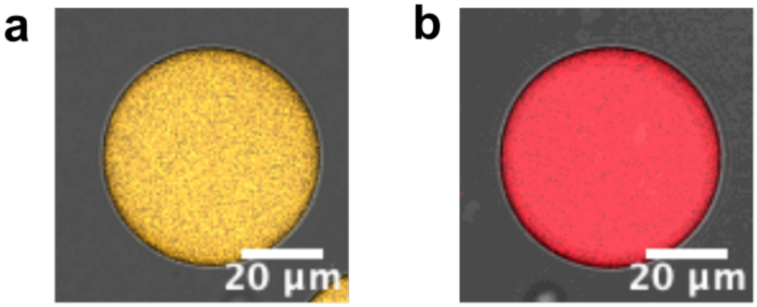

Figure 10: Fluorophore-tagged single-stranded DNA does not interact with droplet-stabilizing surfactants. Representative confocal images of water-in-oil droplets containing Cy3-(**a**, $\lambda_{ex} = 561\,\mathrm{nm}$) and Cy5-labeled ssDNA (**b**, $\lambda_{ex} = 647\,\mathrm{nm}$) without cholesterol-modification at pH8. The solution contained 20 mM potassium phosphate buffer, 10 mM $MgCl_2$ and 1.5 µM DNA. This confirms that there is no unspecific pH-dependent adsorption of the DNA to the droplet periphery at elevated pH. Scale bars: 20 µm.

Supplementary Figure 11: Brightfield and confocal images of microfluidic droplets containing engineered *E. coli*

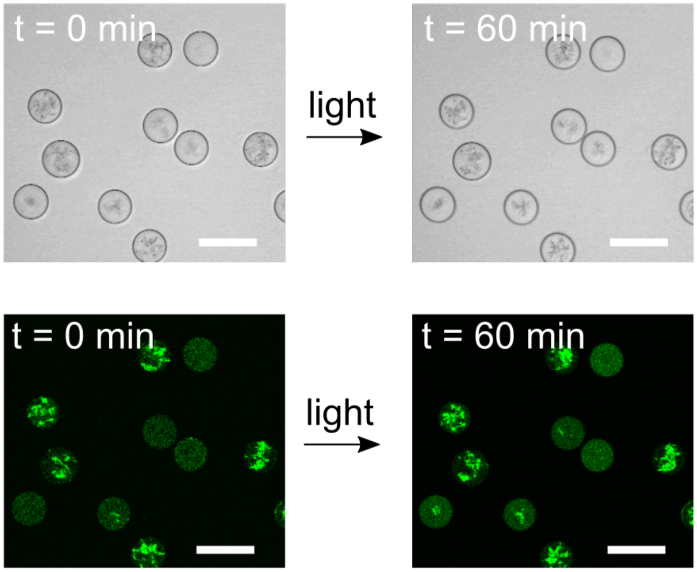

Figure 11: Brightfield (top) and confocal (bottom) images of microfluidic water-in-oil droplets containing the triplex-forming DNA, cholesterol-tagged DNA, pyranine and engineered *E. coli* before (0 min) and after (60 min) illumination with white light. The images confirm the presence of the *E. coli* inside the droplets and their stable confinement. Scale bars: 100 µm.

Supplementary Figure 12: Hysteresis of DNA triplex attachment and detachment

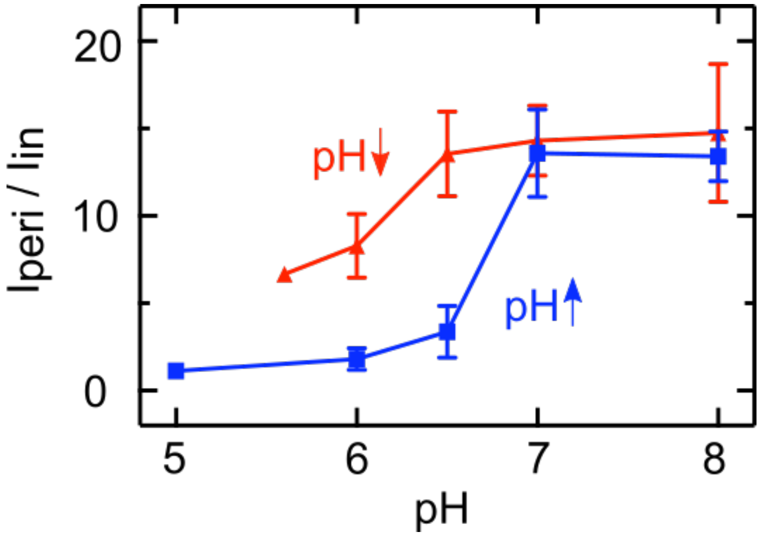

Figure 12: Hysteresis of DNA triplex attachment and detachment. Fluorescence intensity ratio I_{Peri}/I_{In} of the Cy5-labeled triplex-forming DNA strand at the droplet periphery over the droplet lumen at different pH values. The triplex-forming DNA was incubated with the complementary cholesterol-tagged strand for 10 min before encapsulation into droplets with 10 mM sodium phosphate buffer and 20 mM $MgCl_2$ at pH 5 (blue curve) or pH 8 (red curve). After incubation the solutions were mixed 1:1 with 200 mM phosphate buffers ranging from pH 5 to 8. Droplets were then imaged with confocal fluorescence microscopy. The plot clearly indicates that the duplex dissociation happens at lower pH values compared to the duplex formation. This explains why the *E. coli* can induce attachment but not detachment of the triplex-forming DNA. The data depicts mean values and error bars correspond to the standard deviation of n=26 droplets for pH 5, n=57 for pH 6, n=47 for pH 6.5, n=35 for pH 7, n=16 for pH 8 (blue curve) and n=10 droplets for pH 5.6, n=13 for pH 6, n=32 for pH 6.5, n=40 for pH 7 and n=29 for pH 8 (red curve).

Supplementary Figure 13: Cadnano design of the membrane-sculpting DNA origami

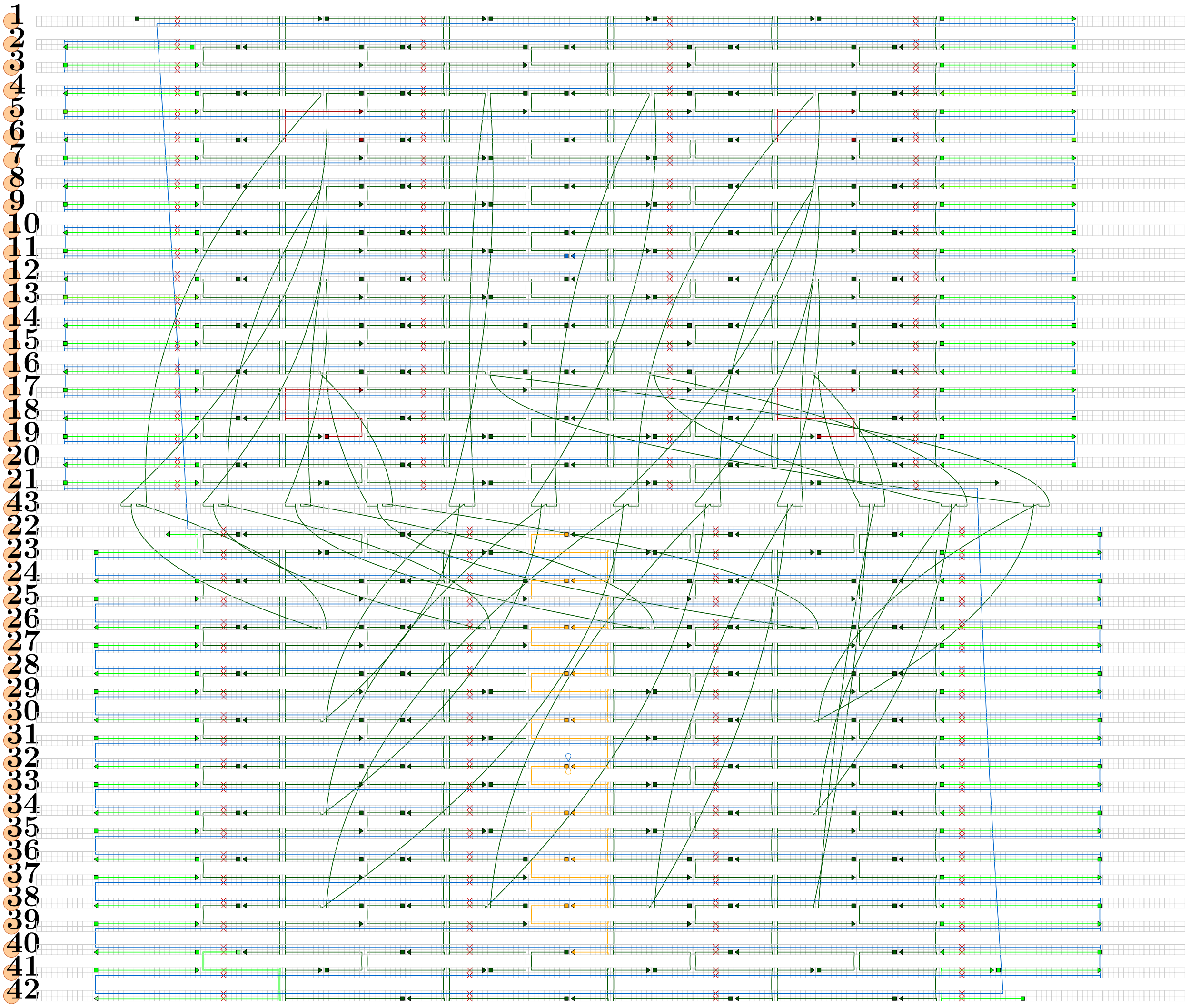

Figure 13: *Cadnano* design of the membrane-sculpting DNA origami. The scaffold (p8064) is shown in blue, bright green staples induce blunt-end stacking, red staples carry overhangs for for the triplex-forming DNA on their 3' end, yellow staples carry overhangs for a complementary Cy3-tagged DNA strand on their 3' end.

Supplementary Figure 14: Blunt-end stacking induces polymerization of DNA origami plates

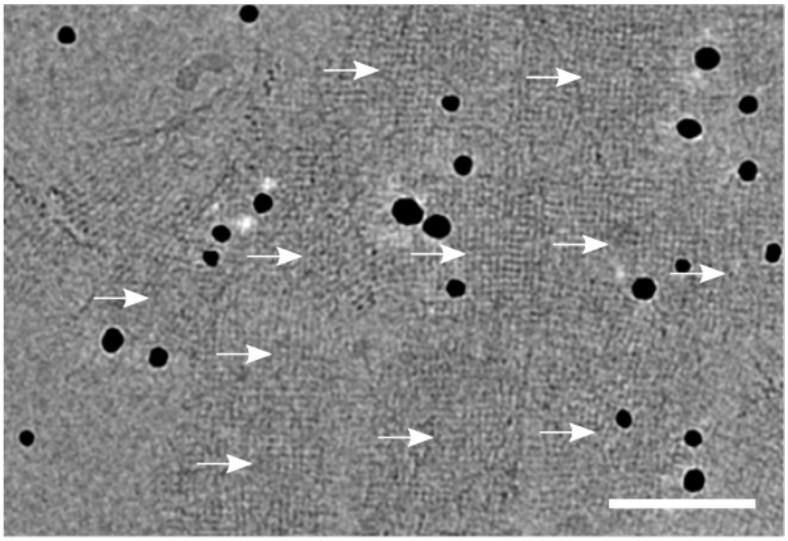

Figure 14: Cryo-EM micrograph of the polymerized DNA origami. The image depicts an arrangement of fused DNA origami squares (indicated by white arrows). Black spots correspond to gold fiducials. This arrangement was used to sculpt the membrane of GUVs. Scale bar: 50 nm.

Supplementary Figure 15: Atomic force microscopy images of the DNA origami

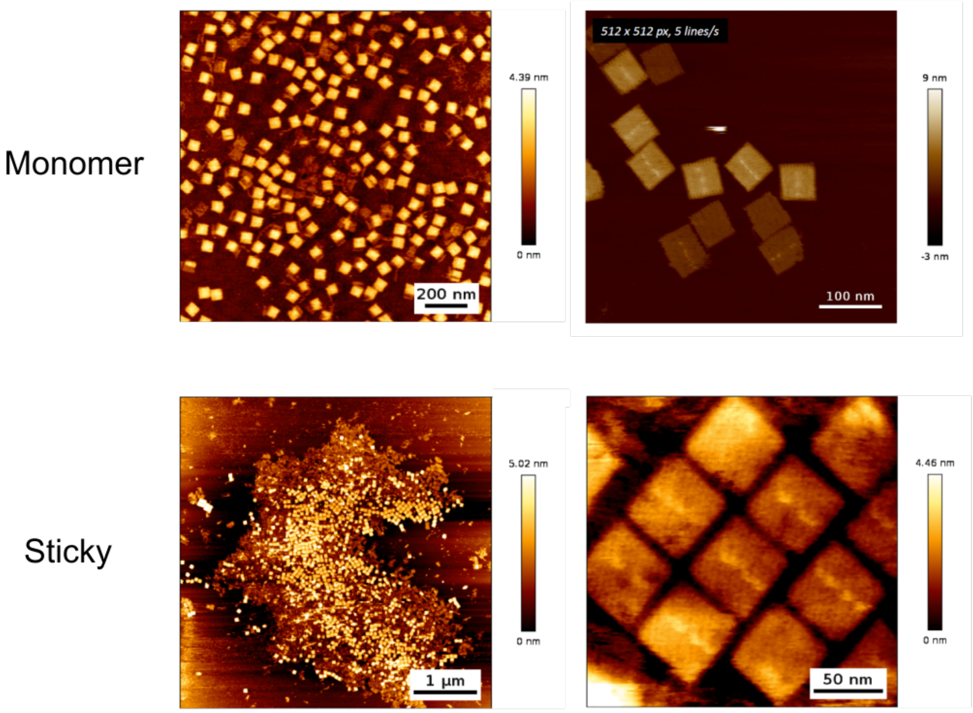

Figure 15: Atomic force microscopy images of the DNA origami without (monomer) and with the overhang strands (sticky). The sticky DNA origami shows a tight packing and cluster formation due to blunt-end stacking, whereas the DNA origami remain loosely distributed when the overhang strands were omitted (monomer). The line that appears across the DNA origami corresponds to the binding sites of the fluorophores. 1 nM of the DNA origami was added to a mica surface, incubated for 90 s and then washed with buffer. Atomic force microscopy was conducted in liquid using a Nanowizzard Ultra Speed 2 (Bruker).

Supplementary Figure 16: Agarose gel electrophoresis of the DNA origami

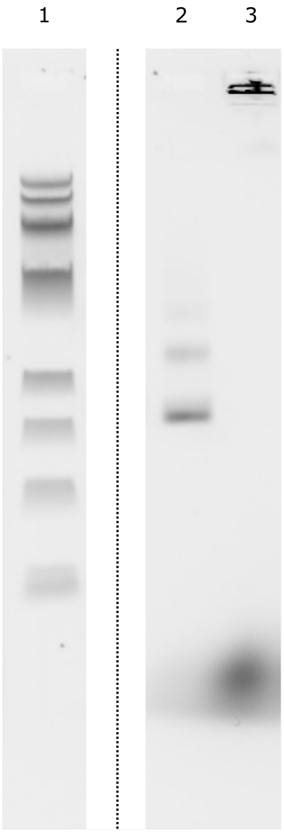

Figure 16: Agarose gel electrophoresis (0.7 % agarose) of the DNA origami. Lane 1) 1 kbp DNA ladder; Lane 2) DNA origami without the staples at the scaffold seam (i.e. with single-stranded scaffold loops); Lane 3) DNA origami with the staples at the scaffold seam. Without the the staples at the scaffold seam (Lane 2), there is a clear band for the monomeric DNA origami and weaker bands from oligomers. The single-stranded scaffold loops prevent blunt-end stacking. In presence of the staples at the scaffold seam, blunt-end stacking occurs and the DNA origami does not leave the pocket due to its highly polymerized state. The gel was run at 60 V for 3.5 h at 4°C.

Supplementary Figure 17: Droplets are not deformed by attaching DNA origami to the droplet periphery

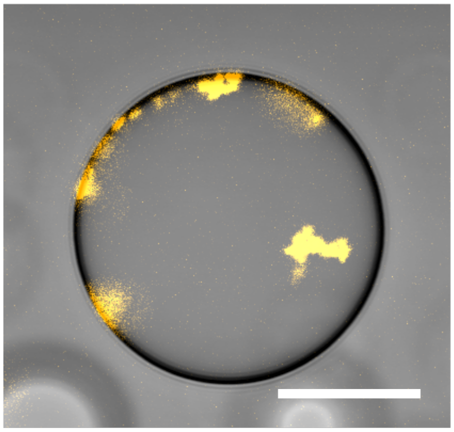

Figure 17: Droplets are not deformed by attaching DNA origami to the droplet periphery. Confocal image of a surfactant-stabilized water-in-oil droplet containing 10 nM cholesterol-tagged Cy3-labeled DNA origami (λ_{ex}=561 nm). The droplet remains spherical even though DNA origami clusters were successfully attached to the droplet periphery. Scale bar: 20 µm.

Supplementary Figure 18: FRAP experiments of GUVs with membrane-bound DNA origami

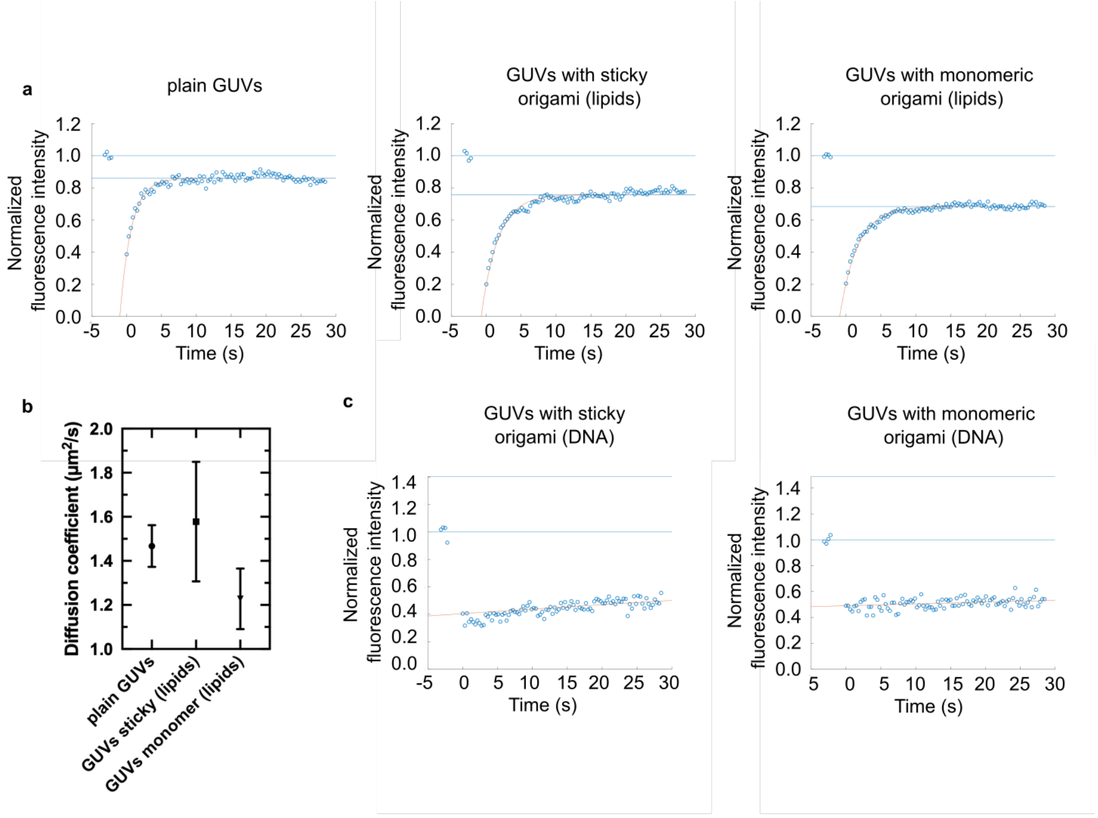

Figure 18: **a** FRAP of lipids (λ_{ex} =488 nm). Exemplary normalized fluorescence recovery after photobleaching (FRAP) traces for plain GUVs, deformed GUVs with membrane-bound polymerized DNA origami (lipid recovery) and spherical GUVs with membrane-bound monomeric DNA origami (with single-stranded scaffold loops, lipid recovery). **b** Diffusion coefficients of lipids in absence and presence of DNA origami (mean ± SD; n=3 for each condition). The lipid diffusivity is not affected significantly by the presence of membrane-bound DNA origami. The diffusion coefficients were calculated according to previous works.[4] **c** FRAP of DNA origami (λ_{ex} =561 nm). Exemplary normalized fluorescence recovery after photobleaching (FRAP) traces for deformed GUVs with membrane-bound polymerized DNA origami (DNA recovery) and spherical GUVs with membrane-bound monomeric DNA origami (with single-stranded scaffold loops, DNA recovery). The DNA origami do not recover after photobleaching as expected in the presence of divalent ions.[5]

Supplementary Figure 19: DNA origami cortex suppresses membrane fluctuations

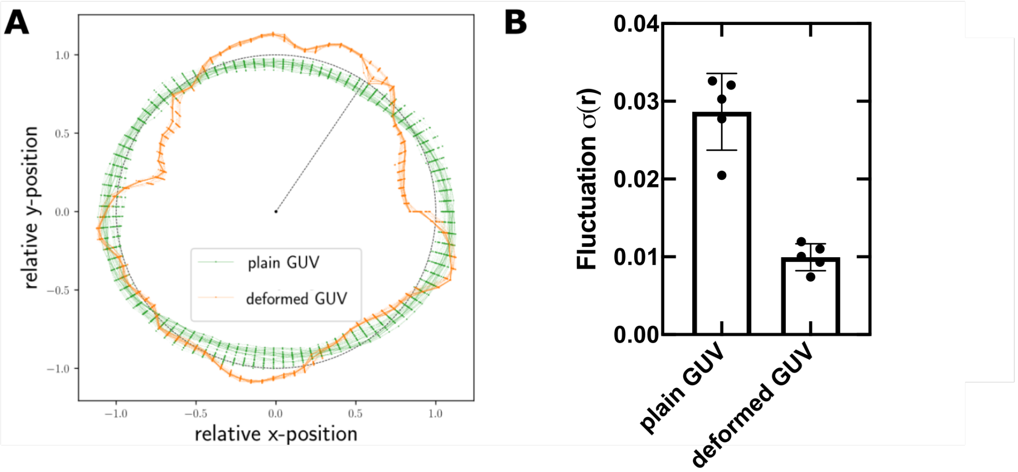

Figure 19: Membrane fluctuations of osmotically deflated GUVs ($c/c_0 = 1.8$) with and without membrane-bound DNA origami. **A** Outline of a plain GUV without membrane-bound DNA origami (green) and a deformed GUV with membrane-bound polymerized DNA origami (orange). The outline was traced from a confocal cross section over time (see also Supplementary Video 5). **B** Standard deviation of the radius r from the mean radius for plain (green) and deformed GUVs (n=5 individual GUVs tracked over time, mean±std.). Higher deviations from the mean radius correspond to larger membrane fluctuations. For the deformed GUV, membrane fluctuations are approximately three times lower than for the plain GUV.

Supplementary Figure 20: Deformation process of GUVs with DNA origami

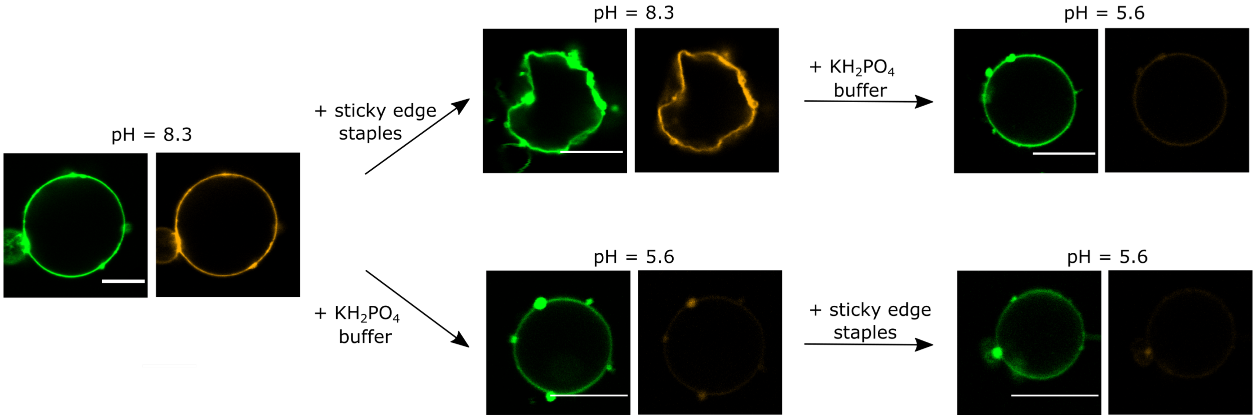

Figure 20: Flow diagram with confocal images of GUVs (lipids labelled with Atto488, λ_{ex}= 488 nm) and DNA origami (labelled with Cy3, λ_{ex}= 561 nm) depicting the deformation process. First of all, we attached the monomeric pH-sensitive DNA origami to the GUVs at pH 8.3 using cholesterol-tags which bind to the hairpin loop of the triplex motif at elevated pH. Subsequently, we added the sticky edge staples, which allow for blunt-end stacking of the DNA origami and thus induce polymerization of the DNA origami. Polymerization, in turn, deforms the GUVs. Following this step, the DNA origami were detached from the GUVs by lowering the pH to 5.6 (upper panels, the fluorescence from the detached DNA origami in the background is too weak to be visible). As a control, we also first lowered the pH to 5.6 and then added the overhang strands (lower panels). This did not affect the morphology of GUVs. After each mixing step, the GUVs were incubated for 24 h either with overhang strands or at a different pH value. Note that the deformation process takes about two hours. Scale bars: 15 µm.

Supplementary Figure 21: Confocal images of deformed GUVs

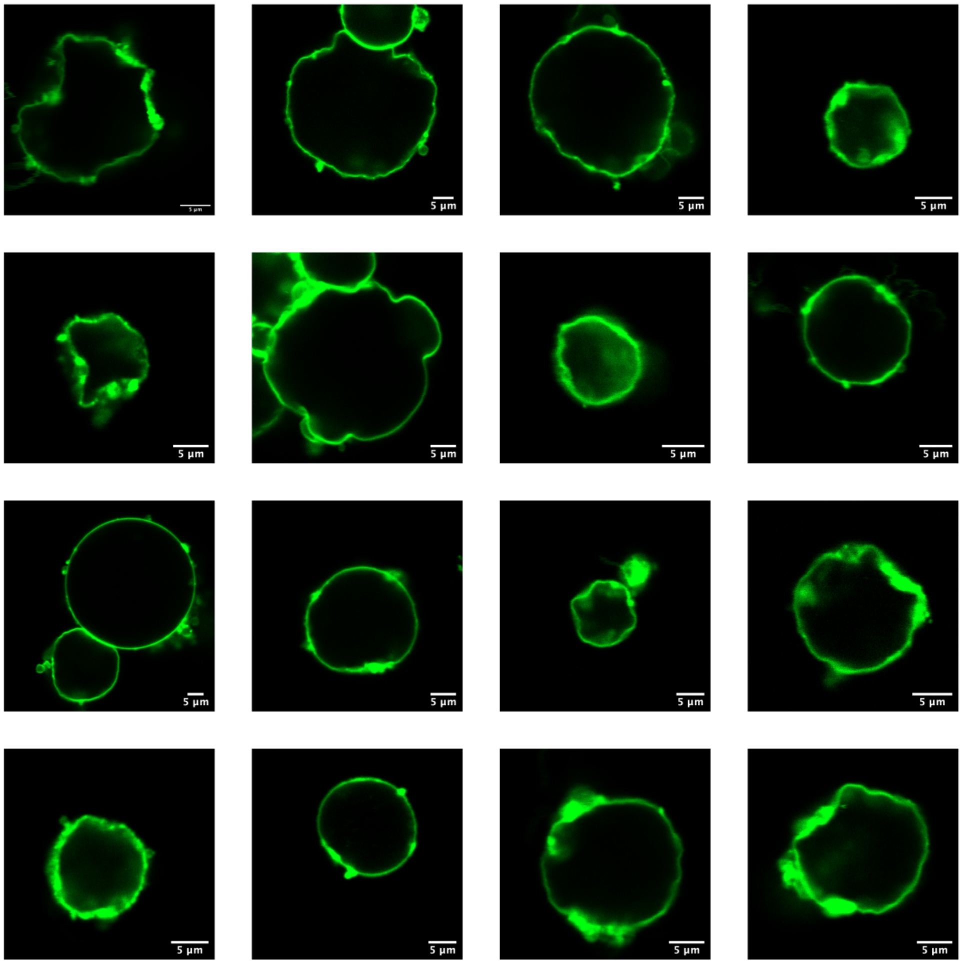

Figure 21: Confocal images of deformed GUVs (λ_{ex}= 561 nm) in presence of membrane-bound polymerized DNA origami at pH 8.3. The corresponding quantification of the GUV circularity is shown in Figure 4e (main text). Scale bars: 5 µm.

Supplementary Figure 22: Confocal images of GUVs after detachment of membrane-bound DNA-origami

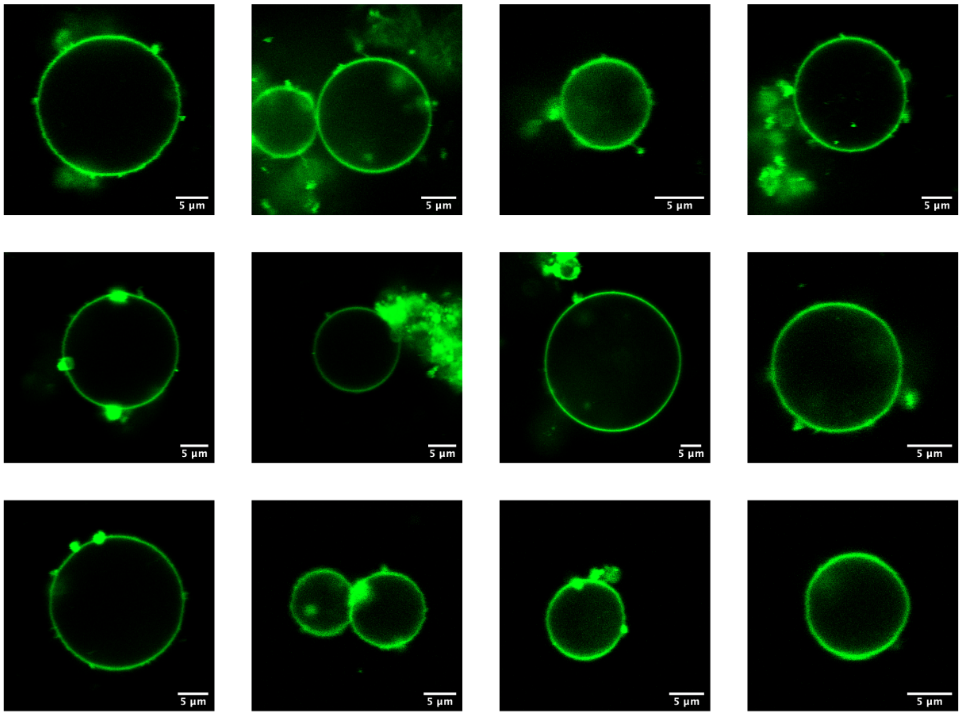

Figure 22: Confocal images of GUVs (λ_{ex}= 561 nm) after decreasing the pH from pH 8.3 to pH 5.6 by addition of iso-osmotic potassium dihydrogenphosphate buffer. The DNA origami detaches from the GUV upon lowering the pH and the GUVs return to a spherical shape. The corresponding quantification of the GUV circularity is shown in Figure 4e (main text). Scale bars: 5 µm.

Supplementary Figure 23: Exemplary confocal images of DNA-functionalized GUVs

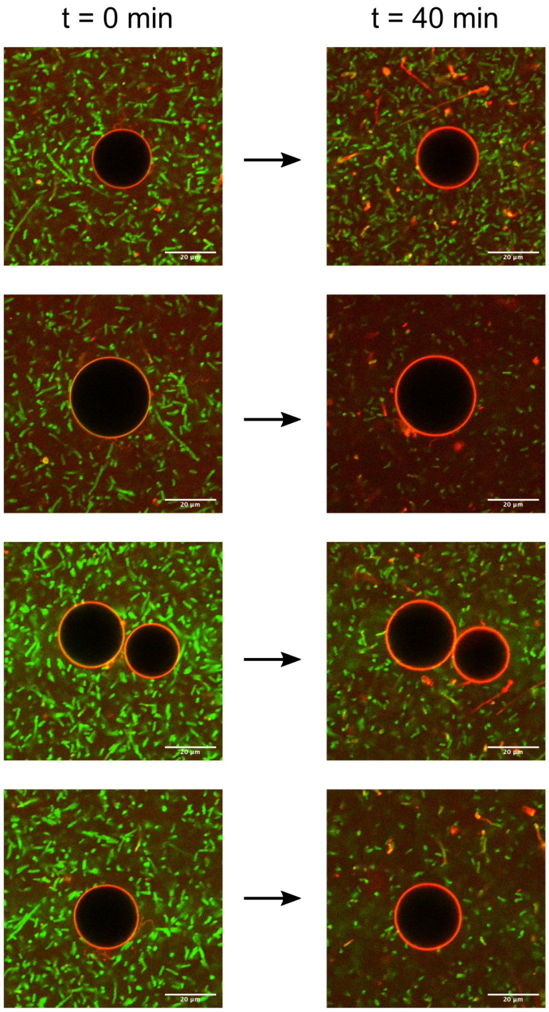

Figure 23: Exemplary confocal images of GUVs coated with cholesterol-tagged DNA (0.6 μM) and surrounded by *E. coli* and triplex-forming DNA (0.4 μM, λ_{ex}=561 nm). The images show the attachment of the triplex-forming DNA after illumination (40 min time point) as well as the settling of *E. coli*. The droplet was illuminated for 15 min after 25 min in the dark. The corresponding quantification of the peripheral DNA intensity is shown in Figure 24. Scale bars: 20 µm.

Supplementary Figure 24: Attachment of the single-stranded DNA triplex to GUVs during light illumination

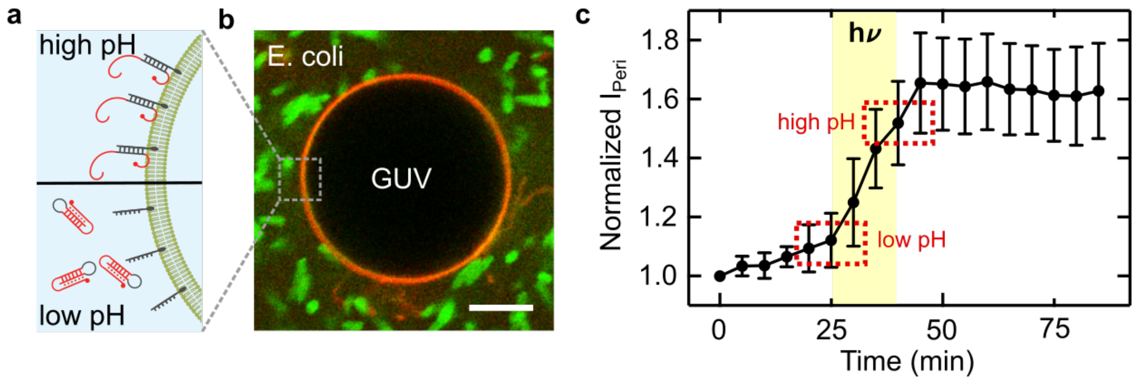

Figure 24: **a** Schematic illustration of a GUV membrane functionalized with cholesterol-tagged DNA in presence of triplex-forming DNA at high and low pH values. **b** Confocal image of a DNA-coated GUV surrounded by *E. coli* as described in **a** (0.4 μM triplex-forming DNA, λ_{ex}=561 nm; 0.6 μM cholesterol-tagged DNA). Scale bar: 10 µm. **c** Normalized fluorescence intensity I_{peri} (mean±s.d., n=15) of the triplex-forming DNA at the GUV periphery monitored over time. The time period of illumination is indicated in yellow, illumination leads to a pH increase and hence DNA attachment. The data is extracted from GUVs as shown in Figure 23.

Supplementary Figure 25: Confocal images of deformed GUVs after light-mediated attachment of DNA origami

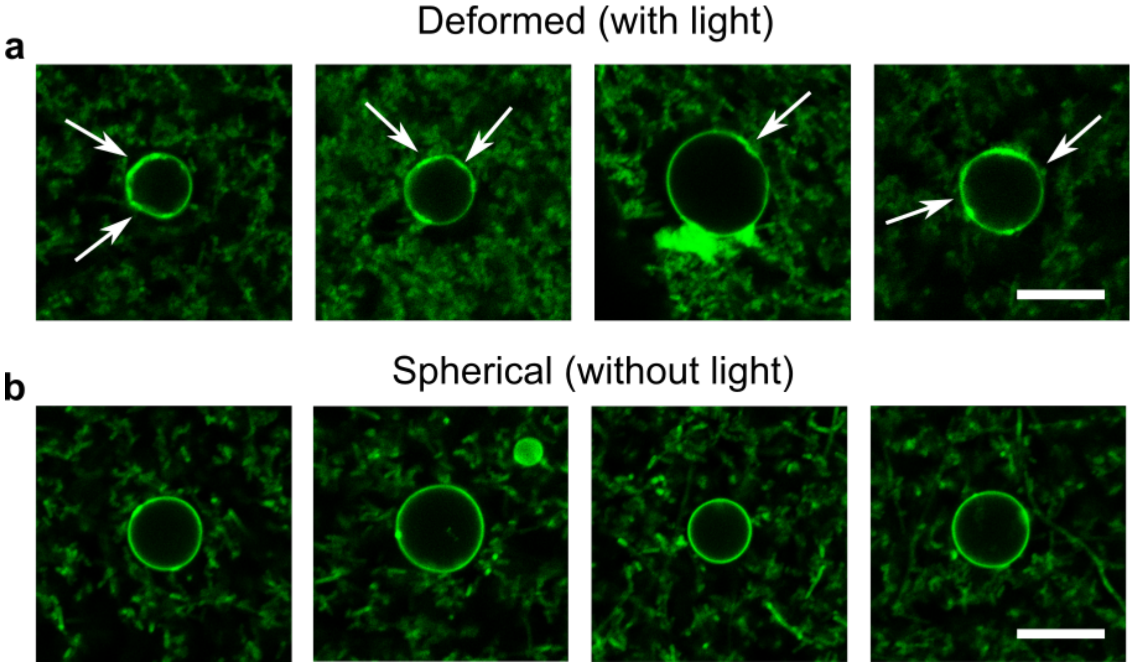

Figure 25: Confocal images of deformed GUVs (λ_{ex}= 488 nm) in presence of membrane-bound polymerized DNA origami and *E. coli* after light-mediated attachment of DNA origami and spherical GUVs without light illumination. Note that the deformation is weaker compared to the deformation achieved with conventional pH switching due to the smaller pH gradient. Scale bars: 10 µm.

Supplementary Figure 26: Light-mediated deformation of GUVs

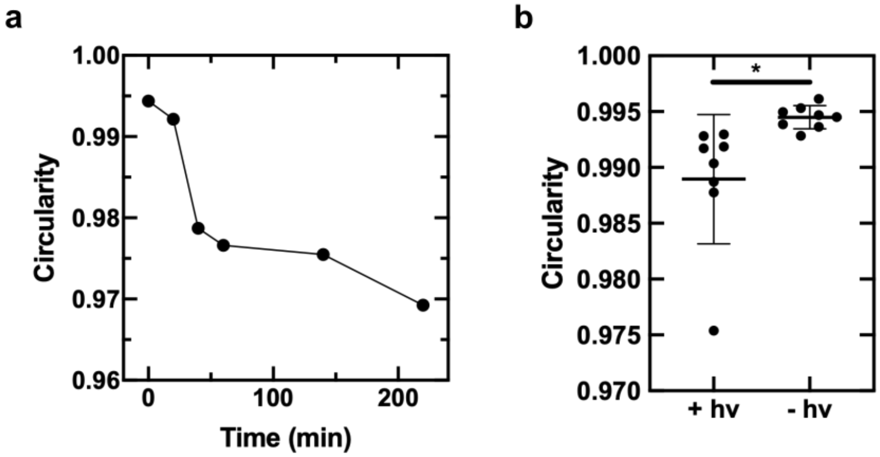

Figure 26: Light-mediated deformation of GUVs. **a** Circularity over time for the representative image of a GUV with attached DNA origami monomers after addition of staple strands at the scaffold seam which enable blunt-end stacking as shown in Figure 5c. The circularity decreases over time corresponding to a deformation of the GUV within 1 h. **b** Circularity of GUVs 14 h after addition of staple strands to the scaffold seam for GUVs that were illuminated with light for 30 min and GUVs that were not illuminated. The circularity for illuminated GUVs is significantly lower than for GUVs that were left in the dark (n=8, error bars show the standard deviation, $p = 0.03$). Note that the density of *E. coli* was $OD_{600} = 20$ for both conditions.

Supplementary Tables

Supplementary Table 1: DNA and amino-acid sequences of xenorhodopsin-constructs

XeR-GFP DNA sequence:

1 ATGGTGTATG AAGCAATTAC CGCAGGCGGT TTTGGTAGCC AGCCGTTTAT TCTGGCATAT
61 ATCATTACCG CAATGATTAG CGGTCTGCTG TTTCTGTATC TGCCTCGTAA ACTGGATGTT
121 CCGCAGAAAT TTGGCATCAT CCATTTTTTC ATTGTGGTTT GGAGCGGTCT GATGTATACC
181 AATTTTCTGA ATCAGAGCTT CCTGAGCGAT TATGCATGGT ATATGGATTG GATGGTTAGC
241 ACACCGCTGA TTCTGCTGGC ACTGGGTCTG ACCGCATTTC ATGGTGCAGA TACCAAACGT
301 TATGATCTGC TGGGTGCACT GCTGGGAGCA GAATTTACCC TGGTTATTAC AGGTCTGCTG
361 GCCCAGGCAC AGGGTAGCAT TACCCCGTAT TATGTTGGTG TTCTGCTGCT GCTGGGCGTT
421 GTTTATCTGC TGGCGAAACC GTTTCGTGAA ATTGCCGAAG AAAGCAGTGA TGGTCTGGCA
481 CGTGCGTATA AAATCCTGGC AGGTTATATT GGCATCTTTT TTCTGAGCTA TCCGACCGTG
541 TGGTATATTA GCGGTATTGA TGCACTGCCT GGTAGCCTGA ATATTCTGGA CCCGACCCAG
601 ACCAGCATTG CACTGGTTGT TCTGCCGTTT TTTTGCAAAC AGGTTTATGG CTTCCTGGAC
661 ATGTATCTGA TTCATAAAGC AGAAGCTCTC GAGGGAGGAA GTCTGGAAGT TCTGTTCCAG
721 GGGCCCGTCG ACGGATCCGA AAACTTGTAT TTCCAGGGCA TGAGTAAAGG AGAAGAACTT
781 TTCACTGGAG TTGTCCCAAT TCTTGTTGAA TTAGATGGTG ATGTTAATGG GCACAAATTT
841 TCTGTCCGTG GAGAGGGTGA AGGTGATGCT ACAAACGGAA AACTCACCCT TAAATTTATT
901 TGCACTACTG GAAAACTACC TGTTCCGTGG CCAACACTTG TCACTACTCT GACCTATGGT
961 GTTCAATGCT TTTCCCGTTA TCCGGATCAC ATGAAACGGC ATGACTTTTT CAAGAGTGCC
1021 ATGCCCGAAG GTTATGTACA GGAACGCACT ATATCTTTCA AAGATGACGG GACCTACAAG
1081 ACGCGTGCTG AAGTCAAGTT TGAAGGTGAT ACCCTTGTTA ATCGTATCGA GTTAAAGGGT
1141 ATTGATTTTA AAGAAGATGG AAACATTCTT GGACACAAAC TCGAGTACAA CTTTAACTCA
1201 CACAATGTAT ACATCACGGC AGACAAACAA AAGAATGGAA TCAAAGCTAA CTTCAAAATT
1261 CGCCACAACG TTGAAGATGG TTCCGTTCAA CTAGCAGACC ATTATCAACA AAATACTCCA
1321 ATTGGCGATG GCCCTGTCCT TTTACCAGAC AACCATTACC TGTCGACACA ATCTGTCCTT
1381 TCGAAAGATC CCAACGAAAA GCGTGACCAC ATGGTCCTTC TTGAGTTTGT AACTGCTGCT
1441 GGGATTACAC ATGGCATGGA TGAGCTCTAC AAAGGAGGAT CTGGTGGTTC TGGGAAGCTT
1501 GCGGCCGCAC TCGAGCACCA CCACCACCAC CACTGA

XeR-GFP amino-acid sequence:

MVYEAITAGGFGSQPFILAYIITAMISGLLFLYLPRKLDVPQKFGIIHFFIVVWSGLMYTNFLNQSFLSD
YAWYMDWMVSTPLILLALGLTAFHGADTKRYDLLGALLGAEFTLVITGLLAQAQGSITPYYVGVLLLLGV
VYLLAKPFREIAEESSDGLARAYKILAGYIGIFFLSYPTVWYISGIDALPGSLNILDPTQTSIALVVLPF
FCKQVYGFLDMYLIHKAEALEGGSLEVLFQGPVDGSENLYFQGMSKGEELFTGVVPILVELDGDVNGHKF
SVRGEGEGDATNGKLTLKFICTTGKLPVPWPTLVTTLTYGVQCFSRYPDHMKRHDFFKSAMPEGYVQERT
ISFKDDGTYKTRAEVKFEGDTLVNRIELKGIDFKEDGNILGHKLEYNFNSHNVYITADKQKNGIKANFKI
RHNVEDGSVQLADHYQQNTPIGDGPVLLPDNHYLSTQSVLSKDPNEKRDHMVLLEFVTAAGITHGMDELY
KGGSGGSGKLAAALEHHHHHH

NsXeR Sf-GFP His_6-Tag

XeR-mCherry DNA sequence:

1 ATGGTGTATG AAGCAATTAC CGCAGGCGGT TTTGGTAGCC AGCCGTTTAT TCTGGCATAT
61 ATCATTACCG CAATGATTAG CGGTCTGCTG TTTCTGTATC TGCCTCGTAA ACTGGATGTT
121 CCGCAGAAAT TTGGCATCAT CCATTTTTTC ATTGTGGTTT GGAGCGGTCT GATGTATACC
181 AATTTTCTGA ATCAGAGCTT CCTGAGCGAT TATGCATGGT ATATGGATTG GATGGTTAGC
241 ACACCGCTGA TTCTGCTGGC ACTGGGTCTG ACCGCATTTC ATGGTGCAGA TACCAAACGT
301 TATGATCTGC TGGGTGCACT GCTGGGAGCA GAATTTACCC TGGTTATTAC AGGTCTGCTG
361 GCCCAGGCAC AGGGTAGCAT TACCCCGTAT TATGTTGGTG TTCTGCTGCT GCTGGGCGTT
421 GTTTATCTGC TGGCGAAACC GTTTCGTGAA ATTGCCGAAG AAAGCAGTGA TGGTCTGGCA
481 CGTGCGTATA AAATCCTGGC AGGTTATATT GGCATCTTTT TTCTGAGCTA TCCGACCGTG
541 TGGTATATTA GCGGTATTGA TGCACTGCCT GGTAGCCTGA ATATTCTGGA CCCGACCCAG
601 ACCAGCATTG CACTGGTTGT TCTGCCGTTT TTTTGCAAAC AGGTTTATGG CTTCCTGGAC
661 ATGTATCTGA TTCATAAAGC AGAAGCTCTC GAGGGAGGAA GTCTGGAAGT TCTGTTCCAG
721 GGGCCCGTCG ACGGATCCAT GCATAGCAAG GGCGAGGAGG ATAACATGGC CATCATCAAG
781 GAGTTCATGC GCTTCAAGGT GCACATGGAG GGCTCCGTGA ACGGCCACGA GTTCGAGATC
841 GAGGGCGAGG GCGAGGGCCG CCCCTACGAG GGCACCCAGA CCGCCAAGCT GAAGGTGACC
901 AAGGGTGGCC CCCTGCCCTT CGCCTGGGAC ATCCTGTCCC CTCAGTTCAT GTACGGCTCC
961 AAGGCCTACG TGAAGCACCC CGCCGACATC CCCGACTACT TGAAGCTGTC CTTCCCCGAG
1021 GGCTTCAAGT GGGAGCGCGT GATGAACTTC GAGGACGGCG GCGTGGTGAC CGTGACCCAG
1081 GACTCCTCCT TGCAGGACGG CGAGTTCATC TACAAGGTGA AGCTGCGCGG CACCAACTTC
1141 CCCTCCGACG GCCCCGTAAT GCAGAAGAAG ACCATGGGCT GGGAGGCCTC CTCCGAGCGG
1201 ATGTACCCCG AGGACGGCGC CCTGAAGGGC GAGATCAAGC AGAGGCTGAA GCTGAAGGAC
1261 GGCGGCCACT ACGACGCTGA GGTCAAGACC ACCTACAAGG CCAAGAAGCC CGTGCAGCTG
1321 CCCGGCGCCT ACAACGTCAA CATCAAGTTG GACATCACCT CCCACAACGA GGACTACACC
1381 ATCGTGGAAC AGTACGAACG CGCCGAGGGC CGCCACTCCA CCGGCGGCAT GGACGAGCTG
1441 TACAAGAAGC TTGCGGCCGC ACTCGAGCAC CACCACCACC ACCACTGA

XeR-mCherry amino-acid sequence:

MVYEAITAGGFGSQPFILAYIITAMISGLLFLYLPRKLDVPQKFGIIHFFIVVWSGLMYTNFLNQSFLSD
YAWYMDWMVSTPLILLALGLTAFHGADTKRYDLLGALLGAEFTLVITGLLAQAQGSITPYYVGVLLLLGV
VYLLAKPFREIAEESSDGLARAYKILAGYIGIFFLSYPTVWYISGIDALPGSLNILDPTQTSIALVVLPF
FCKQVYGFLDMYLIHKAEALEGGSLEVLFQGPVDGSMHSKGEEDNMAIIKEFMRFKVHMEGSVNGHEFEI
EGEGEGRPYEGTQTAKLKVTKGGPLPFAWDILSPQFMYGSKAYVKHPADIPDYLKLSFPEGFKWERVMNF
EDGGVVTVTQDSSLQDGEFIYKVKLRGTNFPSDGPVMQKKTMGWEASSERMYPEDGALKGEIKQRLKLKD
GGHYDAEVKTTYKAKKPVQLPGAYNVNIKLDITSHNEDYTIVEQYERAEGRHSTGGMDELYKKLAAALE
HHHHHH

NsXeR mCherry His$_6$-Tag

Supplementary Notes

Supplementary Note 1: Estimation of the pH change for DNA attachment

In principle, we can quantify the pH change from the quantification of the DNA attachment and the corresponding calibration curve (see Figure 3b). However, the problem in comparing the fluorescent ratios from the calibration measurement in Figure 3b and the DNA attachment mediated via *E. coli* is the fact that the *E. coli* containing droplets absorb some of the excitation as well as emission light from the pH-sensitive DNA which makes us cautious regarding fully quantitative statements on the pH. However, we can still approximate the rise in pH. During light illumination of the droplets with *E. coli* the ratio $I_{\mathrm{peri}}/I_{\mathrm{in}}$ rises from 1.83 to 3.58 and thus by a factor of 1.96. From the confocal images in Supplementary Figure 24 and the pH electrode measurements in Figure 1b, we can deduce the starting point of pH 6.2 before light illumination takes place. This is also in line with the calibration curve in Figure 3b and Supplementary Figure 9, where the DNA starts to attach in between pH 6-6.5. A pH of 6.2 equals a ratio in the calibration measurement of $I_{\mathrm{peri}}/I_{\mathrm{in}}$=1.36 and is thus smaller than the starting value of the measurement with *E. coli* due to absorption of light by *E. coli* on the inside of the droplet and hence a smaller $\mathrm{I_{in}}$. Multiplying the ratio I_{peri}/I_{in}=1.36 at pH 6.2 by 1.96 yields 2.72, which corresponds to a pH of 7.25. This means that by this approximation the pH within droplets rises from pH 6.20 to pH 7.25 during light illumination. This is comparable to the pH range obtained from bulk measurements where the pH increases from pH 6.2 to 7.0 and further in line with a comparison of the images from the calibration measurement with the ones of the droplets after light illumination.

Supplementary Note 2: Estimation of DNA origami density per GUV

To obtain a lower bound estimate for the area coverage of the DNA origami on the GUVs we need to estimate the lipid concentration first. For the electroformation 40 µL of 1 mM lipids in chloroform are spread on the whole ITO slide until all chloroform evaporated. Subsequently, 275 µL of aqueous buffer solution is added into the ring covering $\frac{1}{5}$ of the area covered with lipids leading to a final lipid concentration of 29 µM. Note that this is an upper bound estimate for the lipid concentration since most likely not all lipids will detach from the ITO slide. After mixing the GUV solution with the DNA origami solution the effective concentrations are $c_{\text{lipid}} \simeq 20\,\mu\text{M}$ and $c_{\text{DNA}} = 2\,\text{nM}$. The relative area of the GUV covered with DNA origami is then given by:

$$\alpha = \frac{c_{\text{DNA}} * A_{\text{DNA}}}{2 * c_{\text{lipid}} * A_{\text{lipid}}} = 0.175 \tag{1}$$

with $A_{\text{DNA}} = 250\,\text{nm}^2$ and $A_{\text{lipid}} = 0.7\,\text{nm}^2$. Thus, at least 17 % of the membrane area of the GUVs should be covered with DNA origami.

References

(1) Paxton, W. F.; Price, D.; Richardson, N. J. Hydroxide ion flux and pH-gradient driven ester hydrolysis in polymer vesicle reactors. *Soft Matter* **2013**, *9*, 11295.

(2) Platzman, I.; Janiesch, J.-W.; Spatz, J. P. Synthesis of Nanostructured and Biofunctionalized Water-in-Oil Droplets as Tools for Homing T Cells. *Journal of the American Chemical Society* **2013**, *135*, 3339–3342.

(3) Weiss, M. et al. Sequential bottom-up assembly of mechanically stabilized synthetic cells by microfluidics. *Nature Materials* **2017**, *17*, 89–96.

(4) Jahnke, K.; Weiss, M.; Weber, C.; Platzman, I.; Göpfrich, K.; Spatz, J. P. Engineering Light-Responsive Contractile Actomyosin Networks with DNA Nanotechnology. *Advanced Biosystems* **2020**, 2000102.

(5) Kocabey, S.; Kempter, S.; List, J.; Xing, Y.; Bae, W.; Schiffels, D.; Shih, W. M.; Simmel, F. C.; Liedl, T. Membrane-Assisted Growth of DNA Origami Nanostructure Arrays. *ACS Nano* **2015**, *9*, 3530–3539.
