# Supplementary material for: Proton gradients from light-harvesting E. coli control DNA assemblies for synthetic cells
Source: Nat Commun. 2021 Jun 25;12:3967. doi: 10.1038/s41467-021-24103-x (PMC8233306; doi:10.1038/s41467-021-24103-x)
Supplement: Supplementary file 2 — Description of Additional Supplementary Files [file 41467_2021_24103_MOESM2_ESM.docx]

**Description of Additional Supplementary Files**

**Supplementary Movie 1:** Monitoring of the pH change induced by light-harvesting E. coli with pyranine in bulk. Confocal fluorescence time series of xenorhodopsin-overexpressing E. coli mixed with 50 μM pyranine and GUVs. The sample was illuminated for 5 min after 10, 25 and 40 min. During illumination, the pH of the bulk solution becomes more basic, leading to an increase in pyranine emission upon excitation with 488 nm, hence the apparent blinking in the video. Scale bar: 100 μm.

**Supplementary Movie 2:** Monitoring of the pH change induced by light-harvesting E. coli with pyranine in water-in-oil droplets. Confocal fluorescence time series of xenorhodopsinoverexpressing E. coli mixed with 50 μM pyranine and encapsulated into surfactant-stabilized water-in-oil droplets. The sample was illuminated for 5 min after 10, 25 and 40 min. During illumination, the pH of the bulk solution becomes more basic, leading to an increase in pyranine emission upon excitation with 488 nm. Scale bar: 100 μm.

**Supplementary Movie 3:** DNA attachment to the compartment periphery of water-in-oil droplets by light illumination. Confocal fluorescence time series of Cy5-labeled triplexforming pH-sensitive DNA ( λex = 647 nm within microfluidic droplets containing xenorhodopsin-overexpressing E. coli. The light was turned on for 30 min after 30 min in the dark. During light illumination, the triplex-forming DNA attaches to the droplet periphery. Scale bar: 100 μm.

**Supplementary Movie 4:** Reversible binding of triplex-forming DNA to the droplet periphery. A change of the internal pH of the droplets was achieved by flushing a proton donor or a proton acceptor via the oil phase. Left: Confocal fluorescence time series of microfluidic droplets containing 1 μM triplexforming DNA (green, λex = 488 nm) and 1.5 μM cholesteroltagged DNA. The proton acceptor propylamine (1 vol% in HFE) was flushed into the observation chamber. This causes the pH inside the droplets to increase, leading to the binding of triplex-forming DNA to the periphery. Right: Confocal fluorescence images of microfluidic droplets after flushing the propylamine-containing oil phase. To reversibly switch the pH inside droplet-based compartments and to cause unbinding of the triplex-forming DNA from the droplet periphery, the oil phase was modified with trifluoroacetic acid (1 vol% in HFE) and subsequently flushed into the observation chamber. This causes the pH inside the droplets to decrease leading to the unbinding of triplex-forming DNA. All in all, the video confirms the pH-reversible attachment of the triplex-forming DNA to the droplet periphery. Scale bar: 50 μm.

**Supplementary Movie 5:** DNA origami cortex suppresses membrane fluctuations of GUVs. Membrane fluctuations of osmotically deflated GUVs (c/c0 = 1.8) with and without membrane-bound DNA origami. The plain GUV (green, lipids labelled with Atto488) shows significant membrane fluctuations, while for the deformed GUV with membranebound polymerized DNA origami (orange, DNA origami labelled with Cy3) the fluctuations are suppressed. For a quantitative analysis of the fluctuations, see Supplementary Figure 19.

**Supplementary Movie 6:** Light-mediated attachment of triplexforming DNA to GUVs. Exemplary confocal time series of a DNA-coated GUV surrounded by E. coli (0.4 μM triplex-forming DNA, λex=561 nm;0.6 μM cholesterol-tagged DNA). The time lapse shows the DNA attachment over time after light illumination as well as the settling of E. coli. The droplet was illuminated for 15 min after 25 min in the dark. The corresponding quantification of the peripheral DNA intensity is shown in Figure 4e (main text). Scale bar: 20 μm.

**Supplementary Movie 7:** DNA origami attaches to GUVs during light-illumination. Exemplary confocal time series of a GUV surrounded by E. coli ( λex = 561 nm; 0.6 μM cholesterol-tagged DNA). The timelapse shows the DNA origami attachment over time during light illumination. Note that the E. coli settle over time and therefore disappear from the confocal plane. The GUV was illuminated from 35-50 min and from 110-155 min. The corresponding quantification of the peripheral DNA intensity is shown in Figure 5b (main text). Scale bar: 20 μm.

**Supplementary Movie 8:** DNA origami polymerization deforms GUVs. Exemplary confocal time series of a GUV surrounded by E. coli ( λex=561 nm; 0.6 μM cholesterol-tagged DNA). The timelapse shows the DNA origami-mediated deformation over time after light illumination and addition of the DNA staple strands at the scaffold seam which enable blunt-end stacking and thus polymerization of the DNA origami. Scale bar: 20 μm.

**Supplementary Dataset1:** Details of the well positions, names and sequences of the body, edges and overhangs if the DNA origami plate.
